# Impact of Iron Deficiency on the Growth and Bioelectrical Profile of Different Gut Bacteria

**DOI:** 10.1002/mbo3.70015

**Published:** 2025-06-02

**Authors:** Elisa Quarta, Marwane Bourqqia‐Ramzi, David Muñoz‐Rodriguez, María Teresa García‐Esteban, Antonio Murciano‐Cespedosa, Álvaro Mateos González, Francisco José Conejero‐Meca, Juan Lombardo‐Hernandez, Jesús Mansilla‐Guardiola, Simona Baroni, Simonetta Geninatti Crich, Stefano Geuna, Luca Munaron, Deborah Chiabrando, Celia Herrera‐Rincon

**Affiliations:** ^1^ Department of Biodiversity, Ecology & Evolution, and Modeling, Data Analysis & Computational Tools for Biology Research Group, Biomathematics Unit, Faculty of Biological Sciences Complutense University of Madrid Madrid Spain; ^2^ Department of Molecular Biotechnology and Health Sciences, Molecular Biotechnology Center “Guido Tarone” University of Torino Turin Italy; ^3^ Department of Genetic, Physiology and Microbiology, Unit of Microbiology, Faculty of Biological Sciences Complutense University of Madrid Madrid Spain; ^4^ Department of Clinical and Biological Sciences University of Torino Turin Italy; ^5^ Department of Life Science and Systems Biology (DBIOS) University of Torino Turin Italy

**Keywords:** bioelectricity, membrane potential, microbiota‐gut‐brain axis, nutritional deficiency

## Abstract

Scope: Iron deficiency (ID) is the most common nutritional deficiency worldwide, impacting gut bacteria's metabolism and cellular biochemistry, but its effects on the microbiota‐gut‐brain axis (MGB) are poorly understood. Early‐life ID‐related dysbiosis is linked to neurodevelopmental impairments like autism and attention deficit hyperactivity disorder. Studying ID's impact on bacterial signaling can guide interventions to target MGB in iron‐deficient populations. This study examined the responses of *Escherichia coli* (*E. coli*) and *Limosilactobacillus reuteri* (*L. reuteri*) to in‐vitro ID conditions using the iron chelator 2,2’‐Bipyridyl (BP). Methods and Results: We assessed and modeled their growth and cultivability and explored their bioelectric profiles using the voltage‐sensitive dye DiBAC4(3). Results showed differential responses: *L. reuteri*'s growth and cultivability were unaffected by BP, while *E. coli*'s growth rate and cultivability decreased under ID. Additionally, we created a deterministic mathematical model that demonstrated a decrease in the population's average reproduction rate in *E. coli* under ID. Only *E. coli* exhibited an altered bioelectric profile, marked by increased cell depolarization in ID conditions, which was largely rescued upon the addition of a saturating concentration of iron. Conclusion: These findings highlight specific bioelectrical responses in gut bacteria to ID. Understanding this variability is crucial for deciphering the microbiota's role in health and disease, particularly concerning nutritional iron imbalance and bacterial signaling in the MGB.

## Graphical abstract

This study explores how iron deficiency (ID) affects *Escherichia coli* (*E. coli*) and *Limosilactobacillus reuteri* (*L. reuteri*) under laboratory conditions induced by the iron chelator 2,2'‐Bipyridyl (BP). *L. reuteri* showed no significant changes, whereas *E. coli* experienced reduced growth and altered bioelectric properties with increased cell depolarization. A mathematical model confirmed lower reproduction rates in *E. coli* due to ID. These findings underscore the diverse responses of gut bacteria to ID, offer insights into potential links with microbiota‐gut‐brain disorders, and emphasize the need for targeted interventions in iron‐deficient populations.

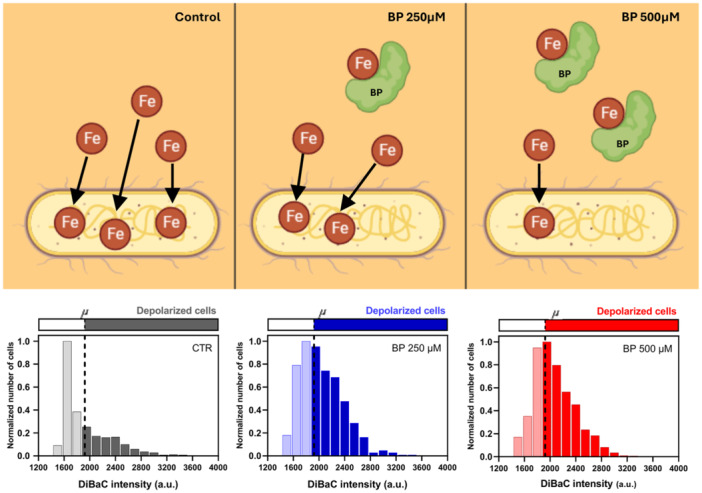

**Abbreviations**
BP: 2,2'‐bipyridyl
CFU: colony‐forming units
CTRL: control group
DiBAC: bis‐(1,3‐dibutylbarbituric acid) trimethine oxonol
FAC: ferric ammonium citrate
ICP‐MS: inductively coupled plasma mass spectrometry
ID: iron deficiency
K^+^
: potassium ion
MGB: microbiota‐gut‐brain axis
MRS: Man Rogosa Sharpe
RT: room temperature
TSA: tripticasein soy agar
TSB: trypticasein soy broth
VBNC: viable but nonculturable
Vmem: membrane potential

## 1 Introduction

Iron deficiency (ID) is the most common nutritional deficiency worldwide, predominantly affecting individuals in low‐ and middle‐income countries, with approximately 1.4 billion people impacted worldwide (Zimmermann and Hurrell 2007). In situations where iron intake fails to meet the body's demands, iron stores become depleted. Absolute ID arises when iron reserves are insufficient to fulfill individual needs, often resulting in anemia. Anemia stemming from low iron levels is especially prevalent among young children (under 5 years old) and premenopausal women, particularly during pregnancy, and is associated with various pathological conditions. The crucial role of iron in systemic cellular biochemistry is widely acknowledged, as it participates in DNA and protein synthesis, acts as a cofactor for numerous enzymes, and contributes to structural proteins and physiological responses (Youdim 2000). Beyond its systemic effects, insufficient iron levels are associated with cognitive and neurobehavioral disorders (Lozoff et al. 2006). Inadequate maternal iron intake during gestation has been linked to autism, schizophrenia, and atypical brain structure in children (Georgieff 2020). Infants with ID exhibit compromised recognition memory, slower processing speed, and poorer bonding, persisting even after postnatal iron repletion (Youdim 2000). Early‐life ID has been shown to induce persistent changes in nociceptive processing (maintained in adulthood). Iron dependence on processes involved in oligodendroglia differentiation and myelination, as well as in dopamine function and related behaviors is extensively reported (Lozoff 2011). Since numerous enzymes in the Central Nervous System (CNS) are iron‐dependent, traditionally, attention has been paid to brain iron metabolism as the major role for the effects of ID on nervous system. Surprisingly, effects of ID on brain function are not fully reversed by iron supplementation therapies, (Felt et al. 2006) highlighting significant knowledge gaps and opportunities to explore the intricate interplay between nutritional ID and brain function.

The question of whether the impact of iron intake on neural tissue is solely direct or if intermediary systems related to diet, such as the gastrointestinal system, influence neural outcomes, remains unresolved. It is evident that dietary iron initially interacts with gut bacteria upon entering the body. While iron is primarily absorbed in the duodenum, any unabsorbed iron can enter the colonic lumen, where numerous microorganisms constituting the gut microbiota (mainly bacteria) reside. Iron is vital for these bacteria, and its availability consequently influences this microbial ecosystem (Seyoum et al. 2021). Iron is generally found in one of the two redox states: oxidized (ferric form) or reduced (ferrous form). Bacterial iron homeostasis is highly regulated, (Andrews et al. 2003) and bacteria use three major strategies to acquire iron: by producing ferric‐specific chelators called siderophores, by absorbing ferrous iron after reducing ferric iron, and by using host iron compounds such as heme and transferrin (Andrews et al. 2013). Prior work has focused largely on the effects of iron fortification (via oral supplementation) on gut ecosystem (Kontoghiorghes et al. 2005) and the serious side effects of iron over accumulation, including an increase in the growth and virulence of gut bacterial pathogens responsible for diarrhea and inflammation (Seyoum et al. 2021; Pasricha et al. 2021). An study by Kostenko et al. recently showed that both iron overload and deficiency significantly alter the fecal metabolome, and that these alterations persist even after a 7‐week washout period where mice returned to a standard diet, highlighting that early‐life dietary iron levels could have prolonged metabolic consequences (Kostenko et al. 2024).

Changes in the gut microbiota induced by nutritional deficiencies have been linked to alterations in the CNS through the microbiota‐gut‐brain (MGB) axis. However, the effects of iron insufficiency on the MGB axis, specifically on the signaling between gut bacteria and neural pathways, are largely unknown. A multitude of studies suggests that the MGB axis may play a pivotal role in the development and progression of certain neurological and neuropsychiatric conditions, including Alzheimer's disease, autism spectrum disorder, depression, and anxiety (as extensively reviewed in Murciano‐Brea et al. (2021)). The association between ID during pregnancy and subsequent microbiome changes in childhood (Dostal et al. 2013) raises an intriguing question: *could ID be influencing gut bacteria, thereby impacting the CNS through the MGB axis?* In exploring a potential interplay between ID and the MGB axis, our initial focus was to investigate the effects of iron‐deficient conditions on two bacterial strains with known differential iron dependencies and that are commonly found in the human microbiota: *Limosilactobacillus reuteri* (*L. reuteri*) and *Escherichia coli* (*E. coli*). *L. reuteri*, along with other species within the Lactobacillus genus, has been shown to be iron‐independent, (Archibald 1983) even improving in some cases the iron adsorption, (Axling et al. 2020) whereas *E. coli* growth is clearly dependent on iron bioavailability. Since we are particularly interested in bacterial signaling to neural pathways, our main objective was to assess whether ID‐induced changes in bacterial growth dynamics might influence potential candidates for signaling to the neural component of the MGB. Specifically, we focused on investigating the effects of ID on a possible mediator of bacterial‐neuronal communication—the bacterial membrane potential (Vmem).

Bacterial cells are able to communicate each other within a population, and with other distant populations, using a sum of signals of different nature (Jones and Larkin 2021). Bacterial membrane displays the ability to establish and maintain gradients of electrical and electrochemical potentials. Previously, it was commonly thought that these Vmem patterns primarily contributed to maintaining bacterial equilibrium, with a primarily homeostatic function (Benarroch and Asally 2020). However, recent research has reshaped this perspective, unveiling the dynamic nature of bacterial Vmem and its involvement in relevant cellular functions and population behavior. Bacterial Vmem changes and subsequent ion fluxes are implied in cell‐to‐cell communication, (Prindle et al. 2015) antibiotic resistance, (Liu et al. 2015) proliferative capability, (Muñoz‐Rodríguez et al. 2023) or environmental sensing (Bruni et al. 2017). Since Vmem is dynamic, and it is used by bacterial cells to interchange biological information, we emphasize the importance of further exploring the dynamics of bacterial Vmem and the bioelectrical changes in response to external stimuli, such as relevant ion deficiencies. Notably, the interaction between bacteria and sensory neurons using ions and ion‐channel mediated signaling, (Chiu et al. 2013) the inter‐bacterial communication via bioelectrical signals within and between biofilms, (Prindle et al. 2015; Humphries et al. 2017) and the bioelectricity‐driven localization of different bacterial species along the gut epithelium (Sun et al. 2024) suggest potential implications for long‐distance bioelectrical signaling within the MGB axis. To address these knowledge gaps and investigate the potential impact of ID on bacterial Vmem as a mediator of bacterial‐neural signaling, we selected two bacterial species with known differences in iron dependency. This choice ensured that any observed effects on Vmem would likely result from ID itself rather than from strain‐specific characteristics. Additionally, Vmem and growth are functionally linked, (Muñoz‐Rodríguez et al. 2023; Bourqqia‐Ramzi et al. 2024) suggesting that ID might induce notable alterations with important implications. To mimic conditions of ID in the bacterial environment, we used the chelator bipyridyl (BP), which has not previously been applied in a model like ours. Therefore, it was essential to confirm that the BP model effectively induced ID in the bacterial strains we studied.

Our research findings indicate that we successfully developed an iron‐deficiency model for bacteria using bipyridyl as a chelator, and *E. coli* and *L. reuteri* responded differently to ID in their culture surroundings. While *L. reuteri* displayed no changes in growth patterns or ability to grow when exposed to ID, *E. coli* experienced noticeable decreases in growth rate and ability to grow under the same conditions. Intriguingly, only *E. coli* exhibited changes in their bioelectric profile under conditions of ID, characterized by increased cell depolarization. The impact of ID on the Vmem in the *E. coli* population was reversed by approximately 90% when the chelator was applied alongside supplemental iron, indicating the clear effects of iron depletion on Vmem patterns. These observations highlight the diverse ways bacterial bioelectrical states react to ID. Given the prominent role of bioelectricity as a potential signaling mediator between neurons and bacteria, understanding these variations is essential for unraveling the microbiota's role in maintaining healthy gut‐brain signaling, particularly in conditions associated with imbalances in iron nutrition that affect the onset and/or progression of neurological disorders.

## 2 Experimental Section

### 2.1 Bacterial Strains and Growth Conditions

The strains used in this study were *Escherichia coli* NCTC 9001 (*E. coli*) and *Limosilactobacillus reuteri* F275 ATCC 23272 (*L. reuteri*). Both strains were stored at −80°C in cryopreservation medium until use. The stock was then used to prepare cultures in liquid media: Trypticasein Soy Broth (TSB, Condalab; Madrid, Spain) for *E. coli* and Man Rogosa Sharpe (MRS, Condalab; Madrid, Spain) for *L. reuteri*. In all experiments, *E. coli* was grown at 37°C with O_2_. *L. reuteri* was grown at 37°C in microaerophilic environment. Microaerophilic conditions were achieved by setting a 1:5 air:culture ratio in the tubes.

### 2.2 Iron Deficiency (ID) Model: 2,2’‐Bipyridyl Iron Chelation

ID conditions were pharmacologically created, using the 2,2’‐Bipyridyl (BP) iron chelator (Sigma‐Aldrich, CAS‐No: 366‐18‐7). BP was prepared in an appropriate volume of ultrapure sterile water to generate a stock solution concentrated 10 mM. Bacterial cells were cultured in their respective culture medium in presence of BP at 250 µM and 500 µM for 8 h. Untreated cells were established as control group (BP 0 or CTR group).

### 2.3 Inductively Coupled Plasma Mass Spectrometry (ICP‐MS)

Intracellular metal quantification (iron, copper, zinc, and manganese) in the two bacteria strains was carried out by ICP‐MS analysis (Element‐2; Thermo‐Finnigan, Rodano (MI), Italy). First, cultures were prepared for cell precipitation. Bacteria were grown as described above. Once the mid‐late exponential phase of growth was reached, the optical density at 600 nm (OD) was measured and adjusted to ~0.01 in fresh TSB and MRS media (for *E. coli* and *L. reuteri*, respectively). The bacteria were cultured for 8 h (h) at 250 µM and 500 µM of BP iron chelator (groups BP 250 and BP 500, respectively), as well as in absence of BP (CTR group). After this, bacteria were centrifuged (4000 g, 20 min, room temperature, RT), washed once in Phosphate Buffered Saline solution (PBS 1X), and centrifuged again (3500 g, 20 min, RT). An aliquot of 0.5 and 2 ml of HNO_3_ (70%) was used to pre‐digest the resulting *E. coli* and *L. reuteri* bacterial pellets, respectively. The final volumes of the pre‐digested samples were measured, and 0.5 ml of each sample was introduced into a microwave digestion system (ETHOS UP Milestone, Bergamo, Italy) with the following heating protocol: ramp up to 150°C in 8 min, followed by 6 min at 150°C. After mineralization, the samples were collected and diluted with an appropriate volume of ultrapure water or HNO_3_ (1%). Calibration curves were obtained using standard metal solutions (Sigma‐Aldrich) in the range 0.2–0.005 μg/mL.

### 2.4 Growth Dynamics Characterization: Optical Density and Cultivability

To evaluate bacterial growth in ID conditions, *E. coli* and *L. reuteri* were grown overnight according to their culture conditions, and sub‐cultured in fresh medium for 12–16 h until OD at 600 nm reached ~0.8, which corresponds to the late exponential phase of growth. At this point, cells were diluted to adjust the sample to an OD ~ 0.01. These cells were growth for 8 h in presence of 250 and 500 µM BP iron chelator or in absence of it (CTR group). A sample of each condition was taken every 2 h (*t* = 0, 2, 4, 6 and 8 h) to measure OD. The growth curve was constructed taking together the OD measurements from three biological replicates (three technical replicates each). To evaluate whether cultivability can be affected by the ID conditions (BP 0, BP 250 µM, BP 500 µM), at each time point (*t* = 0, 2, 4, 6, and 8 h), a sample was taken to dilute from 10^−^^4^ to 10^−^^7^ in PBS 1X and seeded by drop plate method in Petri dishes with Tripticasein Soy Agar (TSA, Condalab, Madrid, Spain) for *E. coli* and MRS for *L. reuteri*. After overnight incubation at 37°C, colony‐forming units (CFU) at appropriate dilution were counted. The growth curve was then constructed taking together the data from three biological replicates with, at least, ten technical replicates for each dilution and each condition. In *E. coli*, the same experiment was conducted to assess a possible rescue of bacterial growth following the administration of the chelator with supplemental iron, in the form of Ferric Ammonium Citrate (FAC; Sigma‐Aldrich, F5879). The experimental conditions included: CTR, BP 500 µM, BP 500 µM + FAC 1 mM, and BP 500 µM + FAC 2.5 mM.

### 2.5 Viability Assay: LIVE/DEAD Baclight Bacterial Viability Kit

Bacterial viability under ID was evaluated by growing *E. coli* and *L. reuteri* in the respective ID conditions (BP 0, BP 250 µM, BP 500 µM) for 8 h. A sample of each condition was taken every 2 h (*t *= 0, 2, 4, 6 and 8 h) to be treated with LIVE/DEAD BacLight bacterial viability kit (Invitrogen by ThermoFisher, L7007) solution. Images were taken with an excitation wavelength of 480‐490 nm and looking at the emission at 500 nm (colored in green, alive cells) and 635 nm (colored in red, dead cells). A Leica DMi8 (Leica microsystems; Milano, Italy) inverted microscope was used. A FITC LP filter was used for an excitation wavelength of 490 nm to detect alive cells (visible in green), while a N2.1 filter was used for an excitation wavelength of 515–560 nm to detect dead cells (visible in red), with an exposure time of 30 ms for each. Paired images of at least ten random fields were taken in each sample, both under FITC LP and N2.1 filters. Percentage of alive cells for each group at each time point was estimated to plot as 100 − ratio red/green.

### 2.6 Bioelectrical Response: DiBAC4(3) Imaging

Patterns of membrane potential (Vmem) in bacteria under the different ID conditions were evaluated using the voltage‐sensitive fluorescent dye bis‐(1,3‐dibutylbarbituric acid) trimethine oxonol or DiBAC4(3) (a Nernstian dye that accumulated in the cell in a potential‐dependent manner; DiBAC; Fisher Scientific ref. B438; Madrid, Spain). Once the mid‐late exponential phase of growth of *E. coli* and *L. reuteri* was reached, the OD was measured and adjusted to 0.01 in fresh TSB and MRS media, respectively. Bacteria grew in the respective ID conditions (BP 0, BP 250 µM, BP 500 µM) until the OD reached ~0.8, which corresponds to the late exponential phase of growth. At this point, cells were centrifuged (2000 g, 10 min, RT) and resuspended in PBS 1X, diluting the sample to an OD ~ 0.3. Due to the outer membrane of *E. coli*, we pretreated the cells with the chelator ethylenediaminetetraacetic acid (EDTA) for loading the fluorescent indicator, as previously described in Kralj et al. (2011) and tested in Bourqqia‐Ramzi et al. (2024) Staining conditions (concentration and incubation time) were selected after an optimization process wherein different DiBAC concentrations and incubation times and temperatures were evaluated for each strain. Cells were incubated with DiBAC 50 µM (for *E. coli*) and DiBAC 100 µM (for *L. reuteri*) for 10 min and 20 min, respectively, at RT in the dark. In *E. coli*, the same experiment was conducted to assess a possible rescue of bacterial bioelectrical response following the administration of the chelator with supplemental iron. The experimental conditions included: CTR, BP 500 µM, and BP 500 µM + FAC 1 mM. A FITC LP filter (Leica DMi8) was used for an excitation wavelength of 450/490 nm with an exposure time of 30 ms. Paired images of at least four random fields were taken in each sample, both in brightfield (BF) and under FITC filters.

### 2.7 DiBAC4(3) Image Analysis

A custom‐written FIJI macro (ImageJ; National Institutes of Health, Bethesda, MD, USA) was used to identify bacterial cells on phase‐contrast images to create a mask for application on the FITC channel, as previously described (Muñoz‐Rodríguez et al. 2023). Noncellular particulates and background noise were eliminated through size filtering and fluorescence intensity for each individual cell was measured. Using the DiBAC average fluorescence intensity from control bacteria (BP 0 or never exposed to iron chelator BP), we set the depolarization threshold at the mean value. Per each replicate and condition, we calculated the percentage of cells above the depolarization threshold from DiBAC images related to BF images. Per each condition, we used three independent biological replicates with three technical replicates and, at least, four images per sample. Histograms were generated by combining data from different replicates of the same condition.

### 2.8 Statistical Analysis

Data obtained from metal quantification, optical density measurements and cultivability assay, were analyzed by one‐way ANOVA (or non‐parametric test Kruskal‐Wallis; factor: metal and time point, respectively), followed by Tukey analysis for multiple comparisons. First, normality and homoscedasticity of the experimental groups (for each metal or for each time point, respectively) were checked by Kolmogorov‐Smirnov test and Barlett test. Bioelectrical response of bacteria (i.e., depolarization) to ID conditions was analyzed using logistic regression models. To do this, the experimental variations in the proportion of depolarized cells among the different experimental groups (increasing concentrations of BP, as a quantitative factor) were evaluated. The statistical analyses used, *p*‐values, and the number of replicate measurements (N) are stated in the Results section, in figure legends and Supplementary Tables. For all cases, a minimum of three biological replicates (including three technical replicates each) were used. Unless otherwise indicated, data are represented as mean ± standard error of the mean (SEM). The significance level was set to 0.05 in all cases. Statistical analysis and graphs were performed using STATA 2017 (Stata Statistical Software: Release 15, College Station, TX, USA) and GraphPad Prism v. 8.0.2. (GraphPad Software Inc., Boston, MA, USA).

### 2.9 Mathematical Model

A birth and death logistic model was used to represent the number of live and dead bacterial cells in *E. coli* population by the time‐dependent density functions n and m respectively. The parameters of the model are *a* birth rate *b*, measured in *h* ^−^^1^, *a* death rate *d*, measured in *h* ^−^^1^, and carrying capacity *K*: the maximal number of bacteria that the system can support. Initial conditions for *n(t* = *0)* = *n*_*0*_ and *m (t* = *0)* = *m*_*0*_ complete the set of model parameters.

Each of the experimental settings considered—CTR group or BP 0, BP 250 μM, and BP 500 μM—is described by a different set of parameters. For a given experimental setting, the system of ordinary differential equations describing *n* and *m* is.

(1) $$\left\{\begin{array}{l} n'(t)=(b-d)\;n\;(t)\;\left(1-\frac{n\;(t)}{K}\right)\\ m\prime \;(t)=\mathrm{dn}(t)\\ n(0)=n_{0},\;m(0)=m_{0} \end{array}\right..$$

The model is described in detail and justified in the Supporting Information. For each experimental setting, the model was fitted to the experimental data via a gradient descent‐type method on least‐square distance as described in the Supporting Information. At each experimental time, data for live and dead bacteria were taken from observations using the LIVE/DEAD BacLight bacterial viability kit.

## 3 Results

### 3.1 ICP‐MS Confirms That 2,2’‐Bipyridyl (BP)‐Treatment Induces Iron Deficiency in *E. coli* and *L. Reuteri*

**Figure 1:**
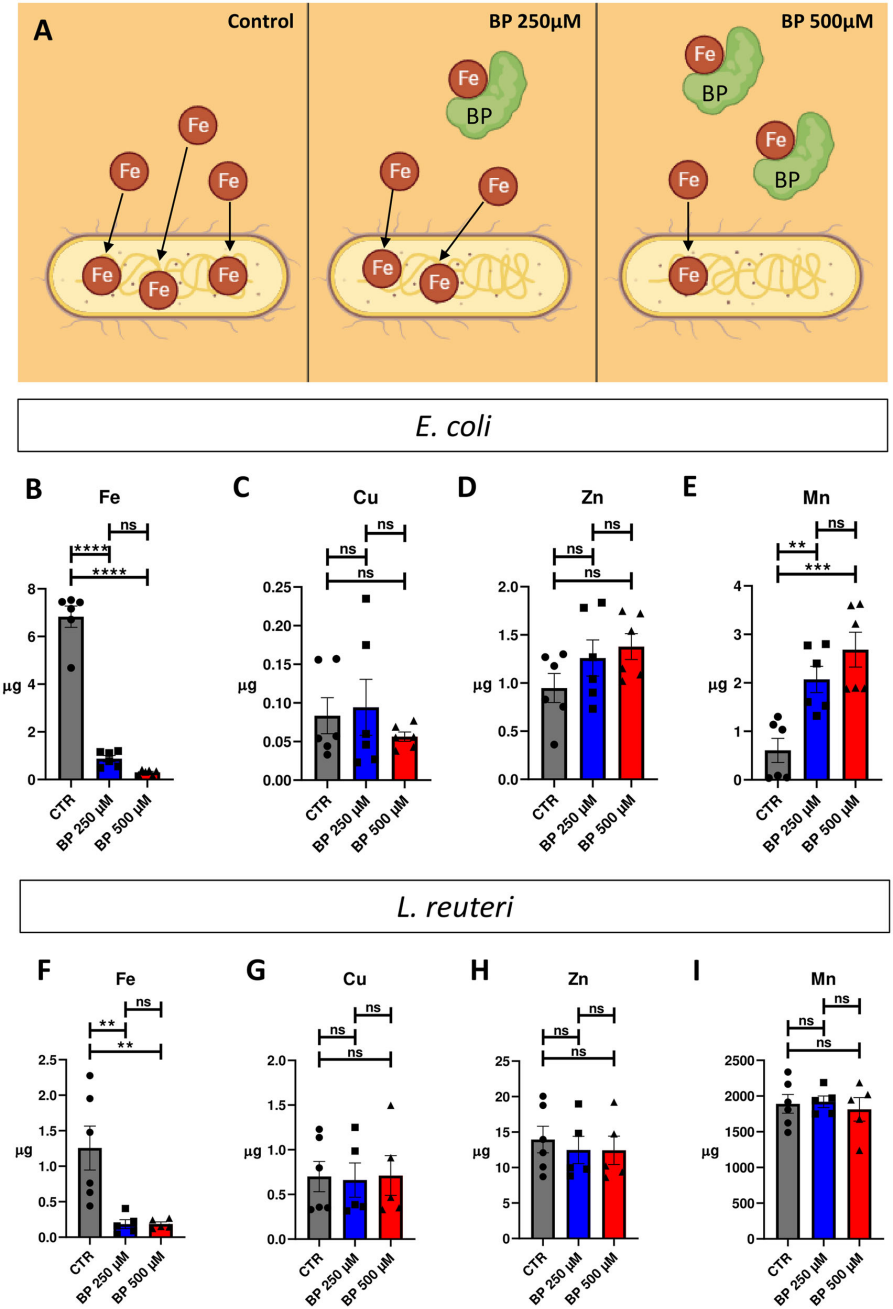
Iron deficient media generation and validation. A. Conceptual schematic showing how 2,2’‐Bipyridyl (BP) is able to create an iron deficient environment in the culture medium which, in turn, causes the decrement of the iron content inside the bacteria. Controlled increasing concentrations of BP are added to the culture medium, in particular concentrations of BP such as 250 and 500 µM have been used. The dimension and steric hindrance of BP doesn't allow it to enter the bacterial cells. Created with BioRender. com. B–E. Histograms showing, respectively, the intracellular amounts of iron, copper (Cu), zinc (Zn) and manganese (Mn) in *E. coli*, comparing Control versus BP 250 µM versus BP 500 µM. F–I. Histograms showing, respectively, the intracellular amounts of iron, copper (Cu), zinc (Zn) and manganese (Mn) in *L. reuteri*, comparing Control versus BP 250 µM versus BP 500 µM. Significance indicated as follows: *p‐value < 0.05, **p‐value < 0.005, ***p‐value < 0.0005.

To induce ID conditions in *E. coli* and *L. reuteri*, we utilized the widely used chelator 2,2’‐Bipyridyl (BP). BP functions as an iron chelator, capable of binding labile iron molecules present in aqueous solutions and sequestering them, thereby creating an iron‐deficient environment (Smith et al. 2021). Consequently, we employed this compound to establish an ID environment in the culture medium for bacterial growth (refer to Figure 1A for a conceptual schematic). To quantify the iron content, we measured the levels of iron in control media without bacteria, Tryptic Soy Broth (TSB) for *E. coli*, and De Man, Rogosa, and Sharpe (MRS) for *L. reuteri*, resulting in concentrations of 7.53 and 4.80 µM, respectively. To confirm the induction of iron deficiency in the bacteria, we utilized Inductively Coupled Plasma Mass Spectrometry (ICP‐MS) to quantify iron and the related metals copper, zinc and manganese (Cu, Zn, and Mn, respectively) in bacterial cells grown under two different concentrations of BP (250 and 500 µM) compared to bacterial cells grown under basal conditions.

In *E. coli*, our results for intracellular iron quantification demonstrated clear differences among bacteria grown in the different ID conditions (Figure 1B). Whereas bacteria grown in control condition contained 6.829 ± 0.447 µg of iron, after BP treatment, this value significantly decreased to 4.789 ± 0.129 and 3.096 ± 0.041 µg for bacteria grown in BP 250 and BP 500 µM, respectively. The decrease in intracellular iron amount induced by BP treatment was proportional to the chelator concentration, but no significant differences were detected between BP 250 and BP 500 µM groups. Conversely, for Cu and Zn quantifications (Figure 1C and Figure 1D, respectively), no differences were found for bacteria grown in absence or in presence of BP, for both two crescent concentrations. For Mn quantification, results demonstrated significant differences among the experimental groups (Figure 1E). CTR bacteria expressed 0.608 ± 0.249 µg of Mn, whereas these values increased up to 1.040 ± 0.271 µg in BP 250 µM and 1.566 ± 0.361 µg in BP 500 µM, with no significant difference in the intracellular Mn between the two BP concentrations (see Supporting Information S1: Table S1 for statistical details).

In *L. reuteri*, our results for intracellular iron quantification demonstrated significant decreases in iron concentration among bacteria grown in the different ID conditions (Figure 1F). CTR bacteria expressed 1.257 ± 0.309 µg of iron, while BP 250 µM‐treated bacteria expressed 0.867 ± 0.063 µg, and BP 500 µM‐treated bacteria expressed 0.459 ± 0.029 µg of intracellular iron. Quantifications of the other metals (Cu, Zn, and Mn) revealed that nonsignificant differences could be detected among bacteria grown in absence or presence of BP (for the two crescent concentrations (Figure 1G–I)).

In summary, our findings confirm the efficacy of BP treatment in inducing discernible and significant reductions in intracellular iron concentration for both *E. coli* and *L. reuteri*. Only Mn demonstrated an increase in *E. coli*, while no significant alterations in other metals were observed in either strain following BP treatment.

### 3.2 Iron Deficiency Impacts Differently on Bacterial Growth: *E. coli Versus L. Reuteri*

**Figure 2:**
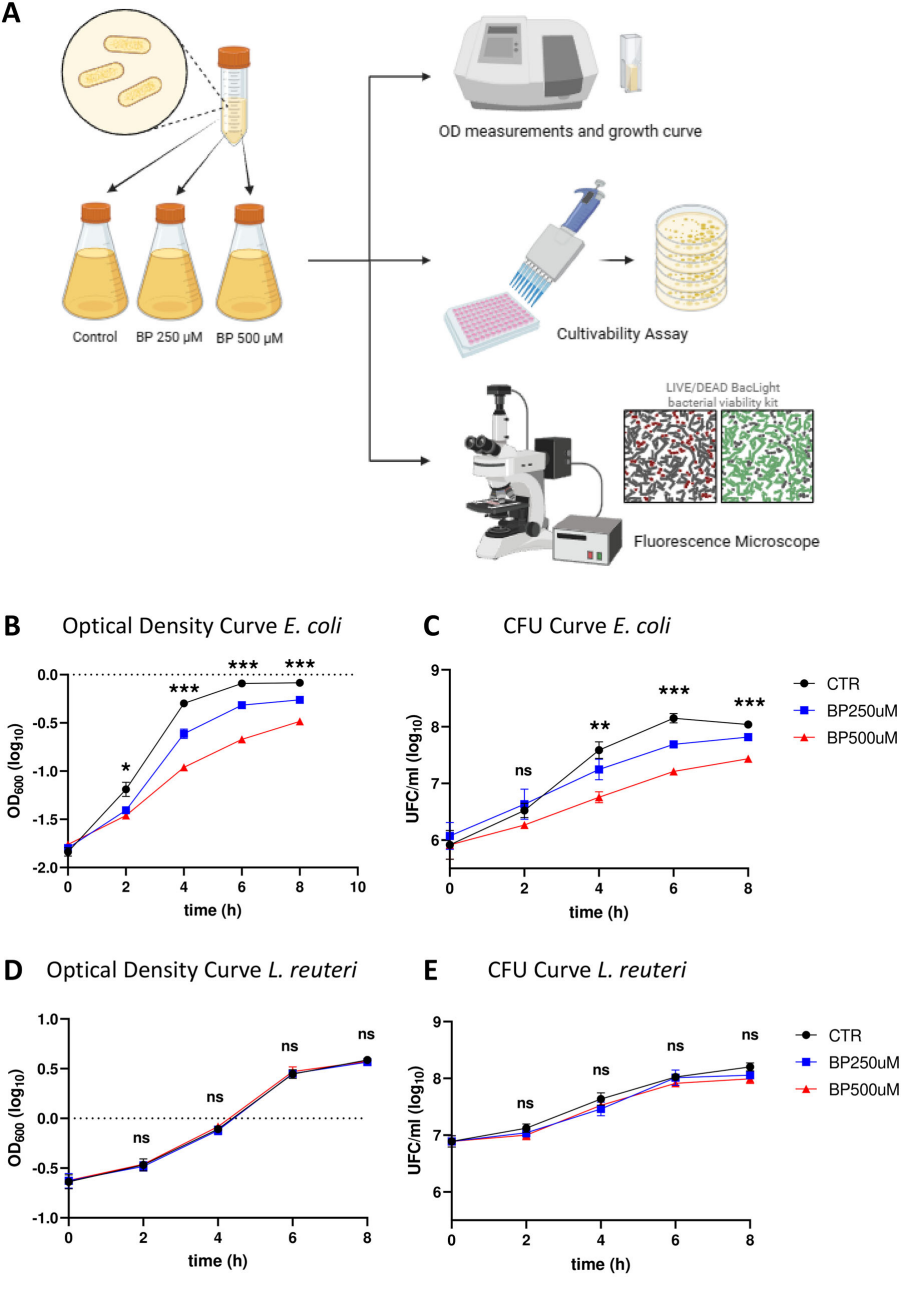
Effects of 2,2’‐Bipyridyl(BP)‐induced iron deficiency on the growth dynamics of different bacterial strains. A*.* Conceptual schematic showing the experimental workflow: bacteria populations were treated with BP for 8 h, taking samples for evaluation each 2 h. Optical Density at 600 nm (OD) measurements, cultivability assays (evaluation of unities able to form colonies when put in solid agar culture medium (colony forming units, CFU/mL)), and microscopic evaluation were evaluated at each time point. B, C. Graphs representing dynamics for *E. coli*. D, E. Graphs representing dynamics for *L. reuteri*. B–D. Data are indicated using a logarithmic scale (log10) on a line plot depicting the average values between three replicates over time (in hours). Control condition (bacterial growing in normal culture medium) is compared to iron deficiency conditions BP 250 µM and BP 500 µM. Different concentrations of BP are indicated as follows: BP 250 µM colored in blue, BP 500 µM in red, no BP treatment (control, CTR) in black. Error bars indicate the standard error of measurement (SEM). Each dot represents the mean value from, at least, three biological replicates with three technical replicates. Significance indicated as follows: *p‐value < 0.05, **p‐value < 0.005, ***p‐value < 0.0005.

Once we confirmed that BP treatment induced deficiencies in intracellular iron in in *E. coli* and *L. reuteri* cells, next we decided to assay the impact of ID on relevant physiological events in both bacteria. To this, we cultivated these bacterial strains during 8 h in the three BP conditions and progressively evaluated the growth dynamics and the cultivability. OD (which is an indicator of the total number of particles that are present inside our sample), cultivability (which measures the number of colony‐forming units, CFU) and viability (ratio of live/dead bacteria) were checked every 2 h (*t* = 0, 2, 4, 6, and 8 h) to timely characterize and graph growth dynamics for each bacterial strain (Figure 2A).

OD measurements in *E. coli* displayed significant differences among the three ID conditions at all time points over the curve (Figure 2B). For each time point, we detected significant differences between CTR group and BP‐treated groups (with a proportional decrease in OD with increasing BP concentrations), but also between the two concentrations of BP treatment, that were becoming significantly different as time passes. Likewise, cultivability in *E. coli* showed a similar pattern (Figure 2C), with increased differences among groups as time progresses. Strikingly, the number of CFU for each condition and every timepoint showed a clear decline when bacteria were grown in ID conditions (respect to CTR group), but also the more BP, and, thus, the more intense ID is, the less cultivability was detected (see Supporting Information S1: Tables S2 and S3 for statistical details). To demonstrate that the effect that we saw in bacterial growth was due to iron deficiency itself and not caused by a toxic effect of BP, we evaluated the percentage of alive cells (using the LIVE/DEAD BacLight Bacterial Viability Kit) comparing pictures taken from the three different BP concentrations (0, 250 µM, 500 µM) at the different timepoints *t* = 0, 2, 4, 6 and 8 h. Interestingly, we detected a similar pattern in the % of alive cells among the three conditions for every time point and, overall, the percentage of alive *E. coli* cells on the total was almost equal to 90% at each timepoint (Supporting Information S1: Figure S1A‐G).

Conversely, OD measurements for *L. reuteri* showed no significant differences among the three BP conditions at all timepoints over the curve (Figure 2D). Multiple comparisons detected no changes between CTR group and BP‐treated groups, neither between the two BP treatments. Cultivability for *L. reuteri* resembled OD pattern (Figure 2E) and no differences in the number of CFU for each condition at every timepoint were detected. To check whether BP was causing any toxic effect on *L. reuteri*, we applied the LIVE/DEAD BacLight Bacterial Viability Kit. Similarly to detected for *E. coli*, the % of alive cells remained unchanged for all cases. Overall, the percentage of living *L. reuteri* cells on the total at different timepoints (time = 0, 2, 4, 6, 8 h) in the three different conditions was higher than 80%–90% (Supporting Information S1: Figure S2A‐G).

**Figure 3:**
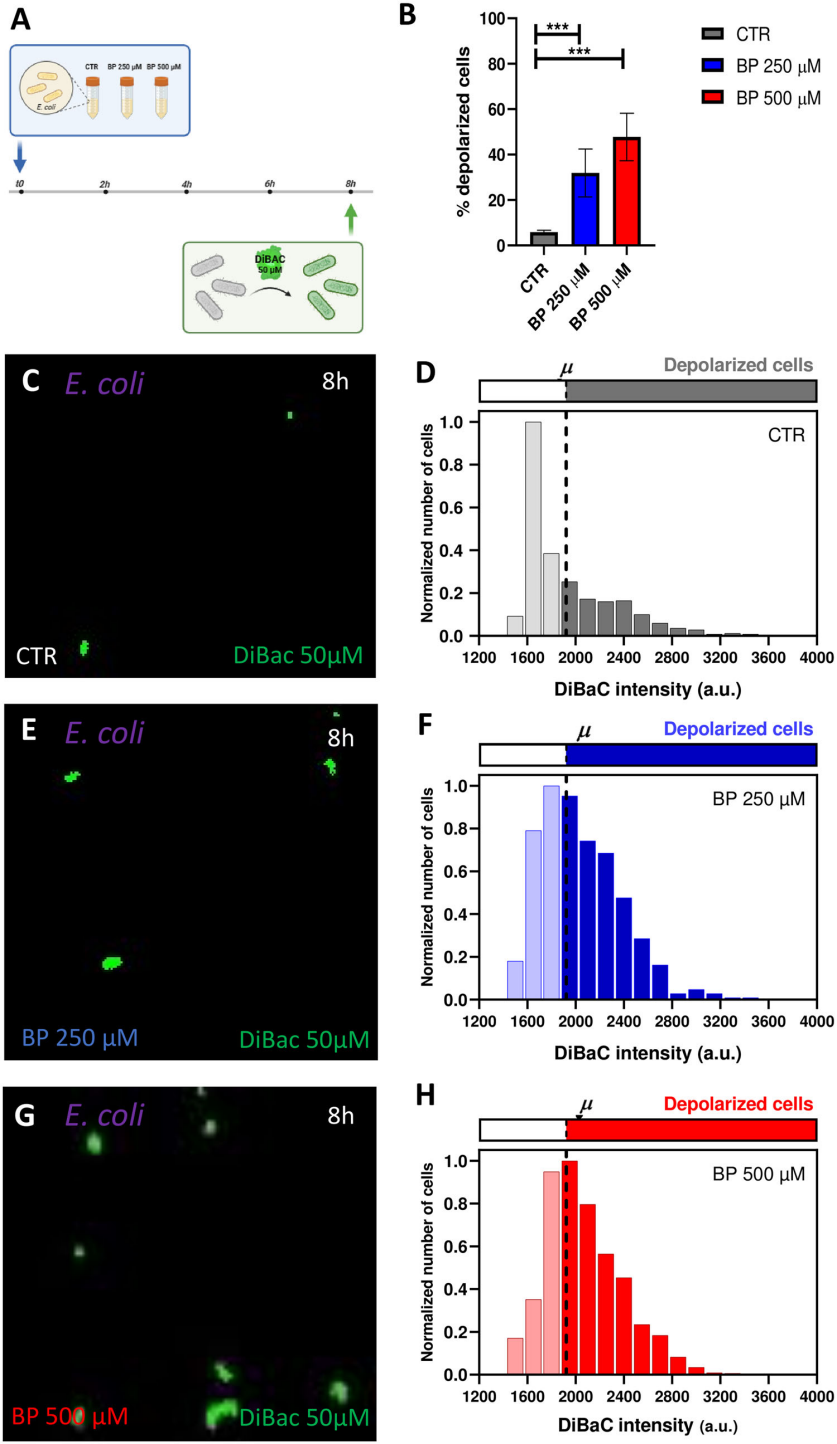
Iron deficiency induces changes in *E. coli* V_mem_ potential. A*.* Conceptual schematic describing the experimental design for the assessment of the depolarization profile in an *E. coli* population. A pure culture of *E. coli* is inoculated in fresh medium and incubated up to > 8 h. At *t* = 8 h bacterial cells are sampled and stained with DiBAC depolarization reporter for the membrane potential (V_mem_) analysis by epifluorescence microscopy. B. Our results revealed a significant increase in the percentage of depolarized cells proportional with BP‐induced iron deficiency. Values from three biological replicates (dots) with three technical replicates, for each condition are represented per time. *p*‐values after applying logistic regression model for percentage of depolarized cells in function of BP concentration are indicated as *** *p*‐value < 0.0005. C, E, G. Epifluorescence microscope images showing DiBAC‐expressing *E. coli* (in green) after 8 h of BP treatment under a 40× objective for CTR (top), BP 250 µM (middle) and BP 500 µM (bottom). The images showed a gradual increasing fluorescent intensity with increasing iron deficiency. D, F, H. Frequency distribution histograms of DiBAC‐expressing *E. coli* cells according to their fluorescence intensity for CTR (top), BP 250 µM (middle) and BP 500 µM (bottom). Data are plotted as the total number of cells, normalized to the number of those exhibiting the most frequent intensity value at each time. Depolarization threshold set at 1922 a.u (dashed line) is calculated as the average DiBAC intensity value of bacterial cells in control condition.

Furthermore, to assess the potential rescue effect of Fe supplementation on growth dynamics, we investigated the growth of *E. coli* following the addition of Ferric Ammonium Citrate (FAC) to the iron‐deficient medium (Supporting Information S1: Figure S3A). We solely conducted this type of assessment in *E. coli*, as *L. reuteri* exhibited no growth dynamics changes in response to iron deficiency. We compared untread cells (CTR) and iron deficiency induced by 500 µM BP, against two different concentrations of FAC added to the ID medium (1 and 2.5 mM). Although some differences in growth were detected between CTRL and BP + FAC‐treated cultures, FAC addition to ID conditions resulted in significant increases in OD measurements at all‐time points over the curve (one‐way ANOVA *p* < 0.001 at 2, 4, 6 h, and *p* < 0.05 at 8 h; Supporting Information S1: Figure 3B, green lines vs red line), therefore leading to a rescue of *E. coli* growth.

Taken together, these results demonstrated that *E. coli* and *L. reuteri* behave differentially in response to iron deficiency in their culture environment: whereas *L. reuteri* showed no alterations in growth dynamics neither cultivability, *E. coli* showed a significant decrease in the growth rate and cultivability upon BP treatment.

### 3.3 Iron Deficiency Affects the Bioelectrical Profile in *E. coli* but Not in *L. Reuteri*

To start exploring the effects of ID on bacterial signaling, next, we decided to study and quantify the bioelectrical characteristics of both *E. coli* and *L. reuteri* under the different BP experimental conditions. To this, we used the fluorescent voltage‐sensitive dye DiBAC as a reliable reporter of Vmem changes in bacterial cells (Muñoz‐Rodríguez et al. 2023; Bourqqia‐Ramzi et al. 2024; Te Winkel et al. 2016) treated with the different BP conditions (Figure 3A). DiBAC is an anionic lipophilic fluorescent probe that enter in the cell when the inner leaflet becomes more positive than the resting Vmem (approx. −90 mV). As cells are more depolarized, more DiBAC enters, and the DiBAC fluorescent signal becomes more intense due to the binding to positively charged intracellular proteins or to the hydrophobic regions (Adams and Levin 2012).

For *E. coli*, our results depicted an increment in the percentage of depolarized cells when bacteria grew in conditions of ID (logistic regression, *p* < 0.0001; Figure 3B). Control cells showed a 5.94 ± 0.88% of depolarized cells (Figure 3C,D), cells treated with BP 250 µM showed a 32.94% ± 10.56% of depolarized cells (Figure 3E,F), and cells treated with BP 500 µM showed a 48.22% ± 10.44% (Figure 3G,H; Supporting Information S1: Figure S4). Statistical analysis revealed significant differences among the three experimental groups (see Supplementary Table S4 for statistical details). Additionally, to get insight into the variability in the bioelectrical profile within the culture, we analyzed the frequency distribution of DiBAC fluorescence intensities per iron condition (Figure 3D,F,H), indicating the depolarization threshold at 1922 a.u. (dashed line) and representing the data normalized to the number of cells whose intensity value was the most frequent at each condition. Our results revealed a clear shift of the curve as iron decreased in the medium, indicating a higher number of bacterial cells with a fluorescence intensity above the depolarization threshold for BP‐treated populations. Moreover, the depolarized fraction at BP 250 and BP 500 µM showed a similar shape, with a greater number of bacteria showing high fluorescence intensity values.

**Figure 4:**
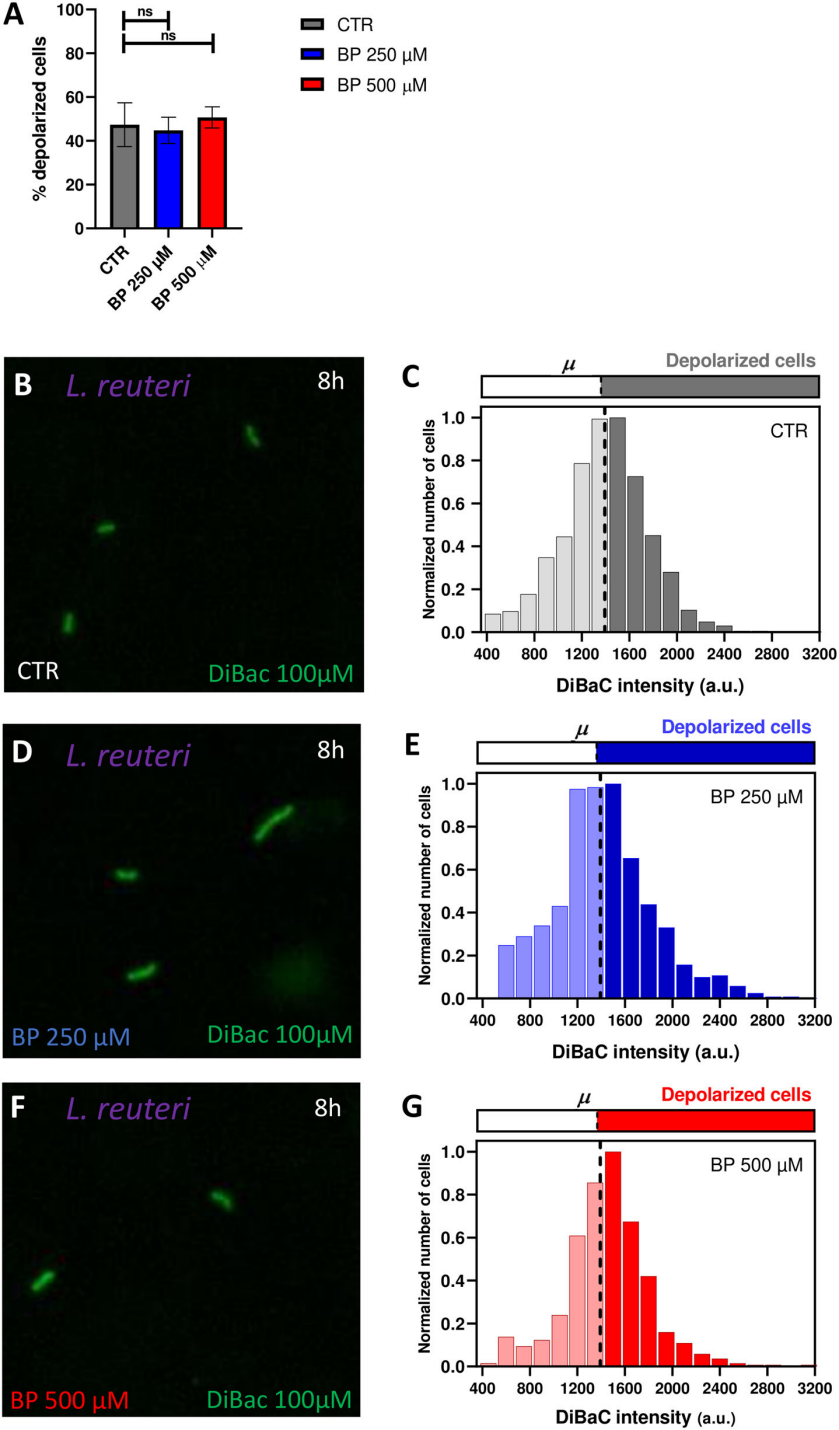
Iron deficiency doesn't induce changes in *L. reuteri* V_mem_ potential. A. Our results revealed no significant change in the percentage of depolarized *L. reuteri* cells proportional with BP‐induced iron deficiency. Values from three biological replicates (dots) with three technical replicates, for each condition are represented per time. p‐values after applying logistic regression model for percentage of depolarized cells in function of BP concentration are indicated as ^ns^ *p* > 0.05. B, D, F. Epifluorescence microscope images showing DiBAC‐expressing *L. reuteri* (in green) after 8 h of BP treatment under a 40X objective for CTR (top), BP 250 µM (middle) and BP 500 µM (bottom). The images showed no significant changes fluorescent intensity with increasing iron deficiency. C, E, G. Frequency distribution histograms of DiBAC‐expressing *L. reuteri* cells according to their fluorescence intensity for CTR (top), BP 250 µM (middle) and BP 500 µM (bottom). Data are plotted as the total number of cells, normalized to the number of those exhibiting the most frequent intensity value at each time. Depolarization threshold set at 1393 a.u (dashed line) is calculated as the average DiBAC intensity value of bacterial cells in control condition.

Conversely, for *L. reuteri*, our results showed similar percentages of depolarization among the three iron conditions (logistic regression, *p* > 0.05; Figure 4A). Control cells displayed a 47.38 % ± 10.04% of depolarized cells (Figure 4B,C), cells treated with BP 250 µM exhibited a 44.78% ± 5.99% of depolarized cells (Figure 4D,E), and cells treated with BP 500 µM presented a 50.71% ± 4.86% (Figure 4F,G; Supporting Information S1: Figure S5). Likewise, the distribution of depolarized cells per intensities (Figure 4C,E,G) displayed similar patterns among groups, with a symmetric bell around the threshold (set at 1393 a.u.) for depolarization.

**Figure 5:**
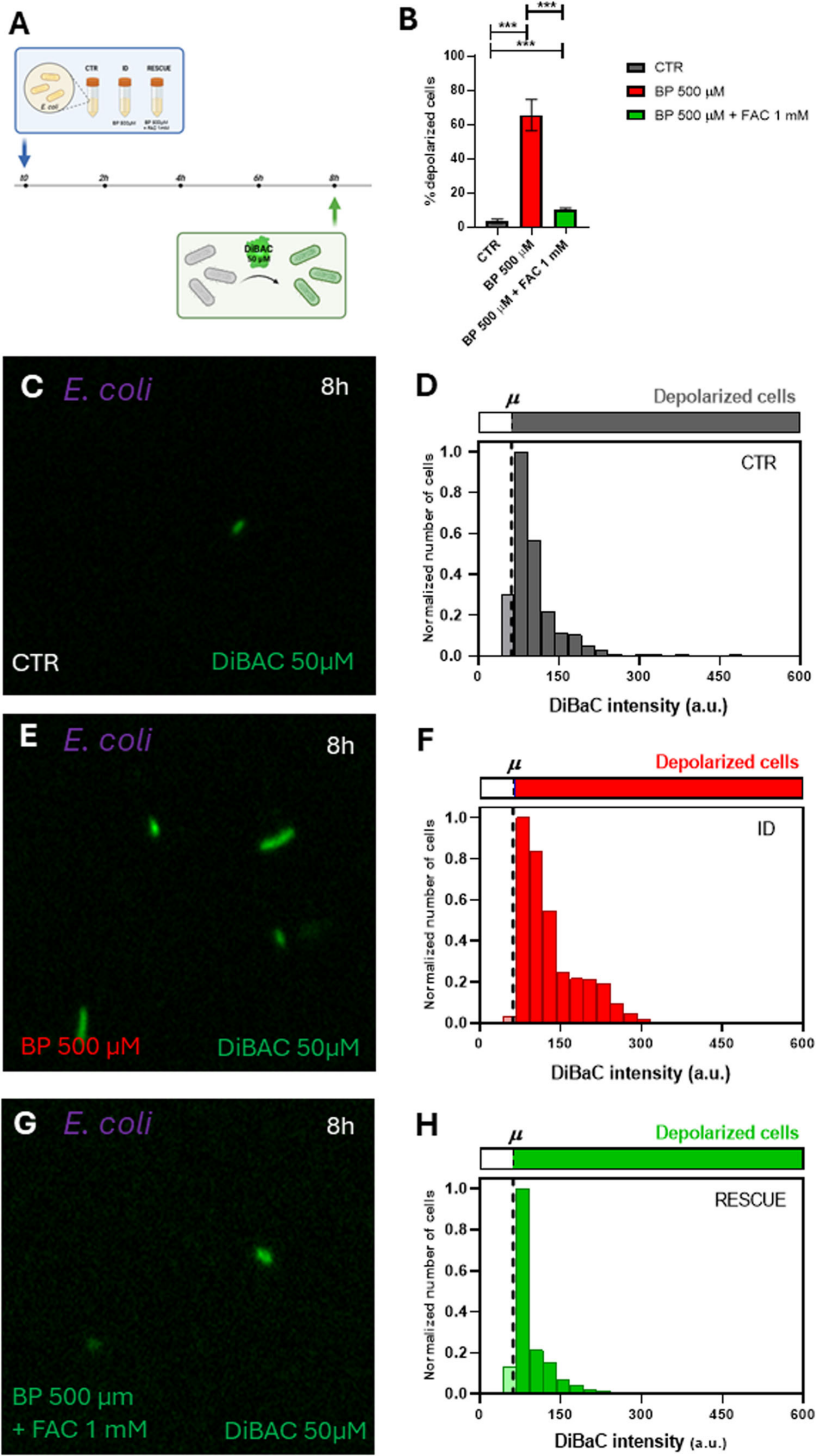
Iron supplementation rescues *V*_mem_ changes in *E. coli* under iron deficiency conditions. *A.* Conceptual schematic describing the experimental design for the assessment of the depolarization profile in an *E. coli* population. A pure culture of *E. coli* is inoculated in fresh medium and incubated up to > 8 h. At *t* = 8 h bacterial cells are sampled and stained with DiBAC depolarization reporter for the membrane potential (Vmem) analysis by epifluorescence microscopy. *B.* Our results revealed a significant increase in the percentage of depolarized cells under BP‐induced iron deficiency, with a notable decrease in depolarization upon iron supplementation (addition of FAC 1 mM). Values from three biological replicates (dots), each with three technical replicates per condition, are presented per time point. *p*‐values after applying logistic regression model assessing the percentage of depolarized cells as a function of BP concentration are indicated as ***p‐value < 0.0005. C, E, G. Epifluorescence microscope images showing DiBAC‐expressing *E. coli* (in green) after 8 h of BP treatment under a 40X objective for CTR (top), BP 500 µM (middle) and BP 500 µM + FAC 1 mM (bottom). The images showed an increasing fluorescent intensity with increasing iron deficiency and a decreasing fluorescent with FAC addition to the iron‐deficient bacteria. D, F, H. Frequency distribution histograms of DiBAC‐expressing *E. coli* cells according to their fluorescence intensity for CTR (top), BP 500 µM (middle) and BP 500 µM + FAC 1 mM (bottom). Data are plotted as the total number of cells, normalized to the number of those exhibiting the most frequent intensity value at each time. Depolarization threshold set at 62.14 a.u (dashed line) is calculated as the average DiBAC intensity value of bacterial cells in control condition.

Given the significant alteration in *E. coli* Vmem patterns under ID conditions, we sought to confirm that these changes were indeed due to iron deficiency and not other effects of the chelator. To test this, we assessed whether adding supplemental iron alongside the chelator would “rescue” the phenotype. Specifically, we analyzed DiBAC fluorescence in *E. coli* following the addition of Ferric Ammonium Citrate (FAC) to the iron‐deficient medium (Figure 5, Supporting Information S1: Figure S6). We compared untreated cells (CTR) and iron‐deficient cells induced by 500 µM BP with those treated with BP 500 µM + 1 mM FAC. Our results showed a significant increase in the percentage of depolarized cells under ID conditions, which decreased in the BP 500 µM + FAC 1 mM condition (logistic regression, *p* < 0.0001; Figure 5B). Control cells displayed 4.49% ± 1.20% depolarized cells (Figure 5C,D), cells treated with BP 500 µM showed 65.56% ± 9.07% depolarized cells (Figure 5E,F), and cells treated with BP 500 µM + FAC 1 mM showed 10.48% ± 0.69% (Figure 5G,H). Statistical analysis revealed significant differences among the three experimental groups (see Supporting Information S1: Table S5 for detailed statistics). To further examine variability in the bioelectrical profile within the culture, we analyzed the frequency distribution of DiBAC fluorescence intensities across iron conditions (Figure 5D,F,H). The depolarization threshold was set at 62.14 a.u. (dashed line), and data were normalized to the most frequent intensity value per condition, providing additional insights into the range of depolarization within the culture.

Overall, these data reveal distinct variations in the bioelectrical profiles of bacteria in response to iron deficiency between the two strains. While the population of *L. reuteri* exhibited no noteworthy changes in the absence of iron, the *E. coli* population displayed a significant reaction to BP treatment. Specifically, there was an observable increase in depolarization as the iron levels decreased in the medium. These ID‐induced changes in Vmem patterns in *E. coli* were reversed by approximately 90% when the chelator was applied alongside supplemental iron.

### 3.4 Predicting Iron Deficiency Effects on Different Bacterial Strains: A Mathematical Model

Considering the significant effects of iron deficiency on *E. coli* population, we asked whether we could mathematically model the iron‐dependent growth in *E. coli* using our experimental data. To this, we performed a least‐squares fit of the logistic model to the averaged experimental data (for a description of the fit procedure and the predicted population growth curves, see Supporting Information). We optimized the fit in each of the experimental conditions—control group, BP 250 μM, and BP 500 μM. We recovered the birth rate *b*, death rate *d*, and carrying capacity of the medium or the number of individuals of a population that can be sustained indefinitely by a given area, *K* (Supporting Information S1: Table S6). The duplication time of the population (in minutes), τ, can be computed from the fitted parameters as

(2) $$\tau =\frac{1}{b-d}\;\ln\;\left(\frac{2\left[K-n_{0}\right]}{K-{2n}_{0}}\right).$$

The recovered parameters indicated that the presence of the BP iron chelator, and the resulting iron deficiency, reduced the population average reproduction rate of *E. coli*. This reduction stemmed from a decrease in the population average birth rate associated with an increase in the population average duration of division time. Moreover, the carrying capacity of the medium appeared to decrease by approximately half upon the addition of a concentration of 250 μM of BP: from the CTR group to the BP 250 μM group, and similarly from the BP 250 μM to the BP 500 μM group. While a concentration of 250 μM of BP appeared to have a negligible effect on the cellular death rate, a higher concentration of 500 μM of BP seemed to amplify the death rate by roughly 2.33 times compared to both the CTR and BP 250 μM groups (Supporting Information S1: Table S7).

In summary, we developed a mathematical model aimed at predicting *E. coli'*s growth patterns under iron deficiency conditions and elucidating the effects of this deficiency on particular parameters of bacterial growth. Our model revealed that reduced population average reproduction rates in iron deficiency conditions are consequence of both a decreased population average birth rate and an increased population average duration of division time.

## 4 Discussion

In this study, we developed an ID model for bacterial culture using the chelator bipyridyl (BP) and demonstrated the effects of BP‐induced ID on different bacterial parameters: growth and bioelectrical state or membrane potential (Vmem) patterns. We chose two bacterial species which exhibit known differential iron dependencies, and that are representative of the human gut microbiota: *E. coli* and *L. reuteri*, Additionally, we introduce a mathematical model that elucidates the behavior of *E. coli* population under iron‐deficient conditions, providing unique parameter estimates for each ID condition. Each bacterial strain responds differentially to ID in terms of growth dynamics: while *L. reuteri* shows no significant changes in growth or cultivability under ID, *E. coli* exhibits decreased growth rate and cultivability. Moreover, only *E. coli* displays an altered bioelectric profile under ID, characterized by increased depolarization of the cell population. Our findings emphasize the dynamic nature of bacterial reactions to ID, highlighting the opportunity for in‐depth study of the resulting changes in bacterial signaling, which can alter the communication within the MGB axis.

Our primary objective was not to examine the effects of ID on bacterial growth itself, but to assess its potential influence on bacterial Vmem. By selecting two strains with differing responses to ID, we aimed to attribute any observed changes in Vmem to ID itself rather than to unique characteristics of each strain. Notably, the functional link between growth and bioelectrical state in bacteria (Muñoz‐Rodríguez et al. 2023; Bourqqia‐Ramzi et al. 2024) supports a detailed analysis of Vmem patterns in bacteria with diverse iron needs. To induce iron deficiency, we exploited a widely recognized iron chelator, 2,2’‐Bipyridyl (BP), which had not been tested in a model like the one presented here, making it crucial to confirm that the BP model successfully induced ID in the bacterial strains under investigation. BP, renowned for its high‐affinity and lipid‐soluble properties, effectively sequesters ferrous iron, thereby replicating conditions akin to those found in the natural environment. The therapeutical efficacy of BP has been demonstrated in various rodent models of cerebral ischemia, where it mitigates brain injury by reducing the concentration of free ferrous iron (Wu et al. 2012). Moreover, compared to other iron chelators like deferoxamine, BP induces a more pronounced iron limitation in a relatively brief timeframe (Cheng and He 2014). Notably, supplementing the bacterial fermentation medium with the iron chelator BP results in exceedingly low iron conditions, prompting a comprehensive reorganization of the gut microbiota (Dostal et al. 2013). Our ICP‐MS results underscore the significant impact of BP in decreasing intracellular iron content in both bacterial strains (Figure 1A,B,F), thus validating the method for mimicking iron deficiency conditions. Bacteria's attraction to iron is derived from its essential role in bacterial growth, comparable to other crucial elements such as carbon, nitrogen, and phosphorus. Iron is involved in various biological processes such as the tricarboxylic acid cycle (TCA cycle), electron transfer chain, biofilm formation, oxidative phosphorylation, nitrogen fixation, and biosynthesis of aromatic compounds. Additionally, iron plays a role in generating metabolic products including porphyrins, toxins, antibiotics, cytochrome, and pigments (Gonciarz and Renslo 2021). Our findings demonstrate that ID differentially affects the growth and cultivability of two different bacterial species (Figure 2). Specifically, the growth of *E. coli* seems to diminish significantly in the presence of BP, leading to a notable reduction in the number of colony‐forming units (CFUs) compared to the control group (Figure 2C). However, despite the presence of iron deficiency, the proportion of viable bacteria relative to the total population remains relatively constant. Throughout all conditions and time points, *E. coli* maintains nearly identical percentages of viable cells, indicating a consistent response to iron deficiency across the experimental conditions (Supporting Information S1: Figure S1). The parameters derived from our mathematical model suggest that ID conditions are acting on the growth by reducing the population average reproduction rate of *E. coli* (see Supporting Information S1: Table S6, Supporting Information S1: Figure S7). According to our model, we observe an increase in the average duration of the cell cycle, which could indicate a slowdown in cellular division. This may occur either uniformly across the entire bacterial population or in a more heterogeneous manner. In the former scenario, there would be a homogeneous decrease in the division rate across all cells. Conversely, in the latter scenario, some bacteria might maintain their metabolic activity while others cease dividing altogether, potentially entering a state known as viable but nonculturable (VBNC). This VBNC population has been the subject of extensive debate in recent literature and has been linked to infectious pathologies (Song et al. 2021). We hypothesize that iron deficiency might trigger a transition between physiological states in the microbiota bacteria, potentially resulting in an increase in the VBNC population. However, our study suggests that this phenomenon is only observed in *E. coli* and not in *L. reuteri*, which remains unaffected by BP administration. Further investigation is warranted to explore this differential behavior and to gain insight into the biological parameters that could be affected the most by the iron limitation, especially those present in our mathematical model (proliferation and death rates, cell cycle duration, and carrying capacity).

The contrasting behaviors observed between these two microbiota species may be attributed to their different reliance on iron internalization from the extracellular environment. *E. coli* appears to strictly depend on this mechanism, rendering it particularly vulnerable to conditions of restricted iron availability. In contrast, *L. reuteri* exhibits greater resilience in the absence of iron, potentially compensating for its scarcity by adapting its metabolism to utilize alternative nutrients. Although iron is crucial for the nutrition of nearly all organisms studied, some notable exceptions have been reported, including *Lactobacillus plantarum* (*L. plantarum*) (Archibald 1983) and *Borrelia burgdorferi* (Troxell et al. 2012). Like our results, ID had no significant effect on *L. plantarum*'s growth rate or extent, indicating either extraordinarily efficient iron acquisition or a very low iron requirement (Archibald 1983). Importantly, *E. coli* demonstrates an adaptive response to limited iron availability by releasing siderophores into the environment. Siderophores are small molecules with a high affinity for iron, secreted by bacteria to chelate and facilitate the internalization of iron (Garénaux et al. 2011). This strategy enables *E. coli* to scavenge iron from its surroundings, (Garénaux et al. 2011; Caza et al. 2011) a capability not observed in *L. reuteri*. The acquisition of iron through siderophores is a crucial aspect of the virulence of pathogenic *E. coli* strains. Indeed, certain pathogenic strains of *E. coli* are known to produce multiple types of siderophores, including enterobactin, salmochelins, yersiniabactin, and aerobactin. Each siderophore exhibits distinct properties and may be regulated differently, providing *E. coli* with the ability to adapt to various environmental conditions. The preference of *E. coli* for extracellular iron has been further supported by research conducted by Appenzeller et al. (Appenzeller et al. 2005) who demonstrated an increase in cultivable *E. coli* cells upon the addition of ferrous sulfate to water with initially low iron concentrations. This finding aligns with our experimental data, as we observe a reduction in *E. coli*'s ability to form colonies under low iron conditions achieved by the administration of BP in the culture medium. Thus, BP could be acting as a siderophore, but it cannot be secreted or internalized by bacterial cells.

*E. coli* is a commensal bacterium which colonizes the gastrointestinal tract of humans, in particular in the mucous layer of the colon, and rarely causes disease except in immunocompromised hosts or where the normal gastrointestinal barriers are breached, allowing this bacterium to colonize a different niche (Kaper et al. 2004). *L. reuteri* is a well‐studied probiotic bacterium which colonizes the gastrointestinal tract, and is able to strengthen the intestinal barrier (Mu et al. 2018). A notable distinction between the two bacterial species under consideration is their Gram staining classification, with *E. coli* being Gram‐negative and *L. reuteri* being Gram‐positive. Previous research has highlighted differences in how Gram‐positive and Gram‐negative bacteria reduce iron (Jing et al. 2021). Specifically, Gram‐positive microbes exhibit ferric reduction when cultured in nitrogen‐rich medium. Interestingly, *L. reuteri* lacks the ability to release siderophores like *E. coli*, but Gram‐positive bacteria possess siderophore‐binding proteins on their membrane. These proteins enable them to internalize iron that is already bound to siderophores without the need for iron reduction (Fukushima et al. 2014). Building on this observation, we hypothesize that the differential growth patterns observed between *E. coli* and *L. reuteri* in iron‐deficient medium may be attributed to morpho‐functional differences in their cell membranes. This discrepancy in membrane composition and structure could influence their ability to acquire and utilize iron, ultimately impacting their growth dynamics under iron‐deficient conditions.

The dynamic changes triggered by iron deficiency in certain physiological aspects of gut bacteria could lead to modifications in how these bacteria communicate with each other and with other cell types. Our findings reveal that iron deficiency‐induced shifts in growth patterns are correlated with alterations in the bioelectrical profiles of *E. coli* (Figure 3). Additionally, *L. reuteri*, whose growth remains unaffected under conditions of iron limitation, exhibits no variations in Vmem patterns (Figure 4), confirming previous findings on the functional coupling between relevant stimuli and changes in bioelectrical properties (Muñoz‐Rodríguez et al. 2023; Bourqqia‐Ramzi et al. 2024). Our data reveal that under ID conditions, *E. coli* population increases the overall depolarization. Of this increase, around 90% was reversed when the rescue treatment with supplemental iron (FAC) was added to the medium (Figure 5), confirming that the primary effect of the chelator is mitigated when iron is supplemented. Bioelectrical profile in bacteria is mainly due to potassium ions (K^+^). The initial onset of iron deficiency may trigger the release of K^+^ ions into the surrounding medium, which subsequently induces cell depolarization. Similar response to nutritional deficit has been detected in a *B. subtilis* biofilm (Prindle et al. 2015). Contrary to being solely a result of changes in the intracellular environment, authors demonstrated that bacteria utilize K+ ion waves, or bioelectrical signaling, to communicate the state of starvation among distant cells throughout the biofilm (Liu et al. 2015; Humphries et al. 2017; Lee et al. 2019). *B. subtilis* and *L. reuteri* populations change their bioelectrical profiles in response to external neural‐type stimuli, such as neurotransmitter drugs (Muñoz‐Rodríguez et al. 2023; Bourqqia‐Ramzi et al. 2024). Specifically, exposure to the neurotransmitters glutamate and GABA induced decreases in the overall culture depolarization, the opposite effect to the increased depolarization detected in *E. coli* under iron limitation. GABA and glutamate could be acting directly on ion channels or receptors in bacterial membrane, while ID could be considered as a trigger signal. Indeed, these dynamic responses in Vmem under different relevant events deserve further investigation to relate with intercellular signaling, as detected in other bacteria (Jones and Larkin 2021). Whether the structural and functional alterations induced by iron deficiency in *E. coli* subsequently translate into changes in how these cells communicate with other cells remains unexplored. Considering the clear evidence relating ID with neurological outcomes, efforts should be put in analyzing potential communication pathways between bacteria and neurons that are affected by iron deprivation. Bioelectrical signals emerge as powerful mediators for bacterial communication. Our results are a proof‐of‐principle and a baseline that demonstrate physiological changes in bacterial bioelectrical profile induced by nutritional deficits in bacterial cells, opening future approaches to prevent and/or revert the consequences of nutritional deficiencies on gut bacteria.

## Author Contributions

**Elisa Quarta:** conceptualization (supporting), data curation (supporting), formal analysis (supporting), investigation (supporting), methodology (lead), validation (lead), writing – original draft (equal). **Marwane Bourqqia‐Ramzi:** formal analysis (supporting), methodology (supporting). **David Muñoz‐Rodriguez:** methodology (supporting). **María Teresa García‐Esteban:** conceptualization (supporting), investigation (equal), methodology (equal), supervision (supporting). **Antonio Murciano‐Cespedosa:** data curation (lead), formal analysis (equal), software (supporting), supervision (supporting), writing – review and editing (supporting). **Álvaro Mateos González:** data curation (equal), formal analysis (lead), supervision (equal), writing – original draft (supporting). **Francisco José Conejero‐Meca:** data curation (supporting), formal analysis (equal). **Juan Lombardo‐Hernandez:** methodology (supporting). **Jesús Mansilla‐Guardiola:** methodology (supporting). **Simona Baroni:** investigation (supporting), methodology (supporting), writing – review and editing (supporting). **Simonetta Geninatti Crich:** investigation (supporting), methodology (supporting), writing – review and editing (supporting). **Stefano Geuna:** conceptualization (supporting), funding acquisition (supporting), supervision (supporting), writing – original draft (supporting). **Luca Munaron:** conceptualization (supporting), investigation (supporting), supervision (supporting), writing – review and editing (supporting). **Deborah Chiabrando:** conceptualization (equal), funding acquisition (supporting), writing – original draft (supporting). **Celia Herrera‐Rincon:** conceptualization (lead), funding acquisition (lead), investigation (equal), project administration (lead), supervision (lead), writing – original draft (lead).

## Ethics Statement

The authors have nothing to report.

## Conflicts of Interest

The authors declare no conflicts of interest.

## Supporting information

Supporting information.

## Data Availability

Further information and requests for reagents may be directed to, and will be fulfilled by, the Lead Contact Celia Herrera‐Rincon (ceherrer@ucm.es).
