# Supplementary material for: Impact of Iron Deficiency on the Growth and Bioelectrical Profile of Different Gut Bacteria
Source: Microbiologyopen. 2025 Jun 2;14(3):e70015. doi: 10.1002/mbo3.70015 (PMC12127773; doi:10.1002/mbo3.70015)
Supplement: Supplementary file 1 — Supporting information. [file MBO3-14-e70015-s001.docx]

**Supporting Information**

**SUPPLEMENTARY INFORMATION**

**S1. Mathematical model of bacterial dynamics in iron-deficiency conditions**

Since the bacterial strain *L. reuteri* did not display significant changes in population dynamics under the effect of iron deficiency, we only model the *E. coli* population in the three different experimental settings we study. Those differ only in initial iron chelator concentration: control, BP 250 μM, BP 500 μM. We will use the same mathematical model to describe the evolution in time of the total population of live bacteria *n(t)* and dead bacteria *m(t)* in each setting, the only difference being that parameters of the model are setting dependent.

However, a fine description of it escapes the scope of our work. We aim to produce a simple deterministic model able to track well enough the concentration of live and dead bacteria over time based on few observations. The simplicity of such a model will allow us to couple it with models for more complex media in further research involving interactions between bacteria and neurons in a way for which a more complex model would not be suitable.

We take a logistic model for the reproduction of live bacteria, following the equation first proposed by Verhulst in the 1830s-40s. Hence, the quantity *bn* $\left( 1 - \frac{n}{K} \right)$ accounts for the reproduction of live bacteria, where the parameter b is named the birth rate of the population, and the parameter Ḱ accounts for medium saturation and eventual iron or nutrient scarcity. We consider the coefficients b and Ḱ to be constant in time, positing that they only depend on the initial concentration of bioavailable iron in our model. This is consistent with our policy of avoiding a description of iron metabolism which is out of the scope of our work.

We understand the logistic congestion effect as a reduction of the birth rate that does not affect the rate of death of bacteria. This is consistent with our optical density observations that indicate that the total number of bacteria, alive and dead, seems to reach a plateau around the end of the experiment, rather than the faster growth of m that would otherwise be observed. Hence, we posit that the death rate of live bacteria d is constant in time, only depending on the initial state of the medium, for the same reasons as above.

Altogether, the hypotheses above yield the following deterministic model:

$$\left\{ \begin{matrix} n' (t) = bn (t) \left( 1 - \frac{n (t)}{Ḱ} \right) \\ \end{matrix} ; t\in[0,\infty) \right.$$

[1]

Defining the carrying capacity K (the maximal number of live bacteria that medium can support)
$K = \frac{b-d}{b}Ḱ$ and the growth rate *r= b-d,* the equation above can be rewritten as follows

$$\left\{ \begin{matrix} n' (t) = rn (t) \left( 1 - \frac{n (t)}{K} \right) \\ \end{matrix} ; t\in[0,\infty) \right.$$

[2]

coupled with the initial conditions n (0) = n_0_, m (0) = m_0_. The system can be solved analytically:

$$\left\{ \begin{matrix} n (t) = \frac{K n_{0}}{n_{0} + [K -n_{0}] e^{-(b - d)t}} \\ m (t) = m_{0} + K \left( \frac{b}{b-d} \right) ln \left( 1 + \frac{n_{0}}{K} [e^{(b - d)t} - 1] \right) \end{matrix} \right.$$

[3]

Other population models would have been reasonable choices to implement, such as a Gompertz model, which is particularly adequate to describe a lag phase in bacteria population dynamics. However, an analogous fitting procedure to the one described in the paragraph below yielded unreasonably high birth rates for the Gompertz model, which led us to prefer the logistic. That may mean that the differences between data sets from different experimental settings seems to be better explained by a reduced reproduction rate and a lower carrying capacity as BP dose increases, rather than differences in lag phase duration.

Fitting procedure

For each experimental setting, for *i* $\in$*{0, 1, 2, 3, 4*, we denote by *n^i^* (respectively *m^i^*) the average over technical replicas with complete data sets of the total count of live (respectively dead) cells at time *t_i_ = 2i* hours. The experimental conditions described in the Methods section guarantee that the initial number of live and dead cells is almost identical in each experimental condition and each technical replica. Hence, we take those initial data as given, and equal to the average of initial data over all replicas in all experimental settings, except one outlier.

For a given experimental setting, we are left with 3 parameters to optimize:

P_j_ = (b_j_, K_j_, d_j_)

where b_j_, K_j_, and d_j_ are the birth rate, carrying capacity and death rate in experimental setting j, j=C representing the control group, j = B1 the BP 250 μM group, and j = B2 the BP 500 μM group.

We define the least-squares cost function.

$$\phi_{j} (P_{j}) = \sum_{i=0}^{4} \left[ {(n_{i}- n_{P_{j}} (t_{i}) )}^{2}+{(m_{i}- m_{P_{j}} (t_{i}) )}^{2} \right]$$

where (n, m) p_j_ are the solutions described in equation (3) for the parameters P_j_.

We fit each of the three experimental settings by minimizing Φ_j_ . We perform the minimization. procedure numerically using Matlab R2023a, using the function *fminsearchbnd* with the initial guess for the parameters *P being.*

*P guess = (1.6, 10^10^, 0.01)*

for every experimental setting. This initial guess is informed by a preliminary exploration of the parameter space, and a heuristic that the birth rate should be lower than $ln (8)\approx2.08$, which corresponds to a cellular duplication time of 20mn in a Malthusian setting (death rate equal to 0, and infinite carrying capacity).

We minimize over a set of admissible parameters by setting lower and upper bounds two orders of magnitude away from the expected values for *K_j_* and *d_j_* and corresponding to duplication times at death rate 0 and infinite carrying capacity between roughly 8.3mn and 416mn - wide bounds that guarantee all realistic parameters are explored:

$$\begin{matrix} 0.1 & \leq b_{j} \leq& 5 \\ {10}^{8} & \leq K_{j} \leq& {10}^{12} \\ {10}^{-4} & \leq d_{j} \leq& 1 \end{matrix}$$

The **Supplementary Tables S6 and S7**, and **Supplementary Figure S7** depict the fitted model and the experimental data with error bars corresponding to the standard deviation both in normal and logarithmic scale for the abscissas. Cellular populations are taken per mL of medium.

**SUPPLEMENTARY FIGURES**

**Supplementary Fig. S1**. **Effect of 2,2’-Bipyridyl on *E. coli* viability.**


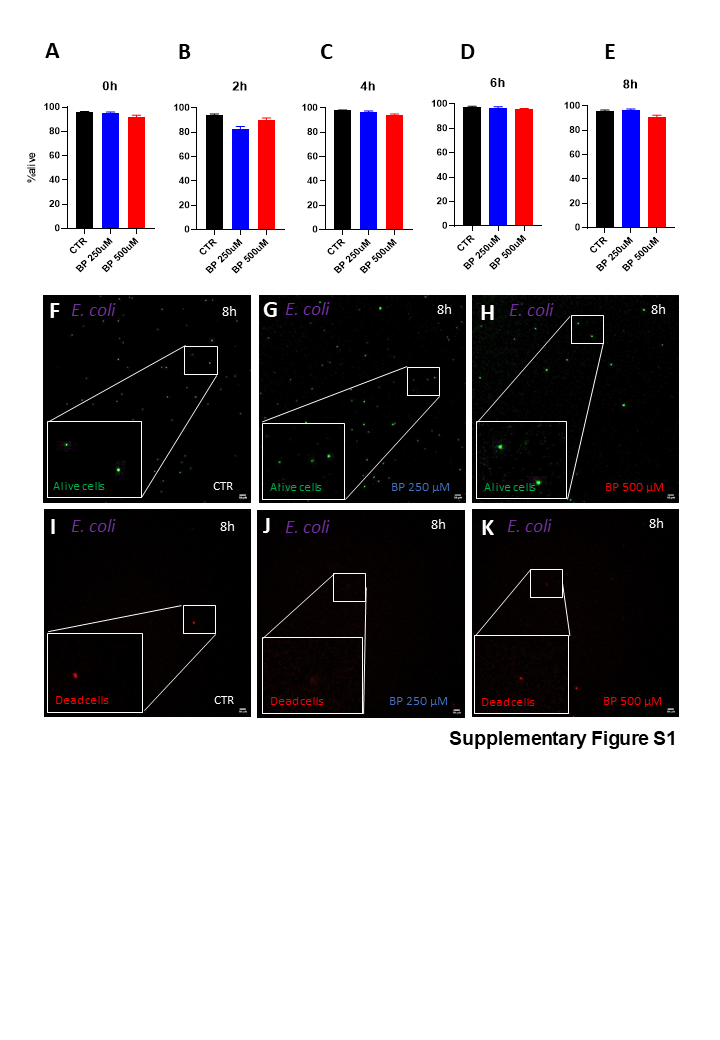


***A-E.*** Histograms showing the percentage of alive *E. coli* cells among the total for t=0, 2, 4, 6, 8 hours. Control condition is colored in black, treatment with BP 250 µM colored in blue, treatment with BP 500 µM colored in red. Bars are indicated with their respectively standard error mean (SEM) calculated from 3 biological replicates with 10 technical replicates each, for each of the three conditions at each timepoint. ***F.*** Picture of *E. coli* alive cells (in green) after 8 hours of normal culture condition (CTR). ***G.*** Picture of *E. coli* alive cells (in green) after 8 hours of treatment with BP 250 µM. ***H.*** Picture of *E. coli* alive cells (in green) after 8 hours of treatment with BP 500 µM. ***I.*** Picture of *E. coli* dead cells (in red) after 8 hours of normal culture condition (CTR). ***J.*** Picture of *E. coli* dead cells (in red) after 8 hours of treatment with BP 250 µM. ***K.*** Picture of *E. coli* dead cells (in red) after 8 hours of treatment with BP 500 µM. All pictures have been taken with Leica DMi8 (Leica microsystems; Milano, Italy) inverted microscope and analyzed with ImageJ.

**Supplementary Fig. S2**. **Effect of 2,2’-Bipyridyl on *L. reuteri* viability.**


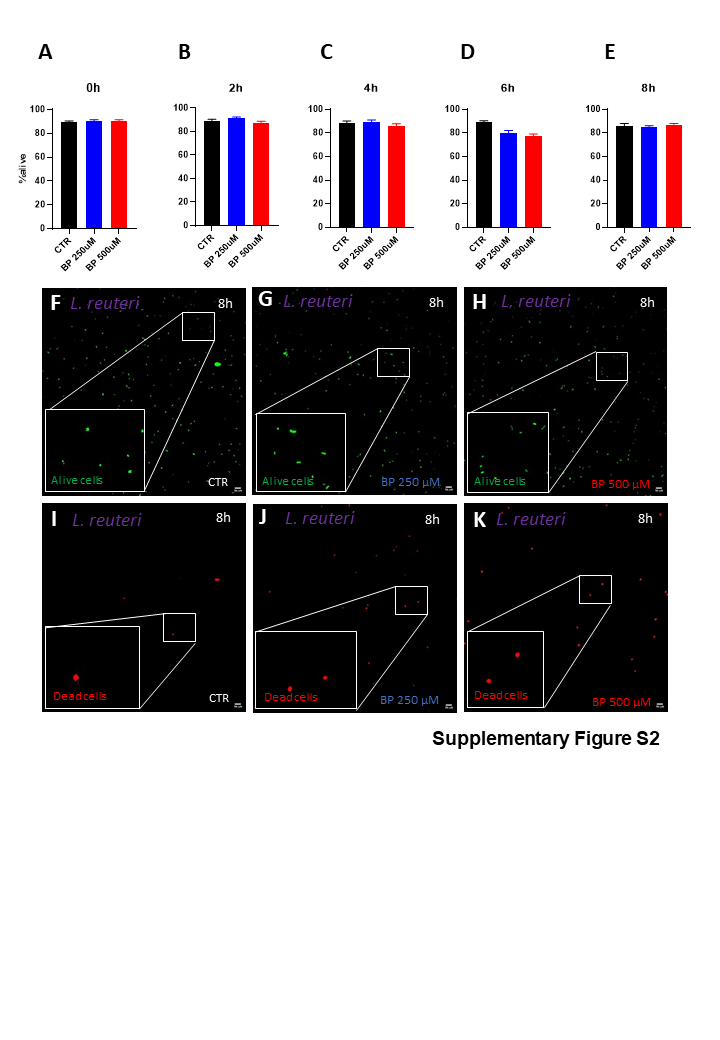


***A-E.*** Histograms showing the percentage of alive *L. reuteri* cells among the total for t=0, 2, 4, 6, 8 hours. Control condition is colored in black, treatment with BP 250 µM colored in blue, treatment with BP 500 µM colored in red. Bars are indicated with their respectively standard error mean (SEM) calculated from 3 biological replicates with 10 technical replicates each, for each of the three conditions at each timepoint. ***F.*** Picture of *L. reuteri* alive cells (in green) after 8 hours of normal culture condition (CTR). ***G.*** Picture of *L. reuteri* alive cells (in green) after 8 hours of treatment with BP 250 µM. ***H.*** Picture of *L. reuteri* alive cells (in green) after 8 hours of treatment with BP 500 µM. ***I.*** Picture of *L. reuteri* dead cells (in red) after 8 hours of normal culture condition (CTR). ***J.*** Picture of *L. reuteri* dead cells (in red) after 8 hours of treatment with BP 250 µM. ***K.*** Picture of *L. reuteri* dead cells (in red) after 8 hours of treatment with BP 500 µM. All pictures have been taken with Leica DMi8 (Leica microsystems; Milano, Italy) inverted microscope and analyzed with ImageJ.

**Supplementary Figure S3. Partial rescue of the growth dynamics of *E. coli* with Ferric Ammonium Citrate (FAC)**.


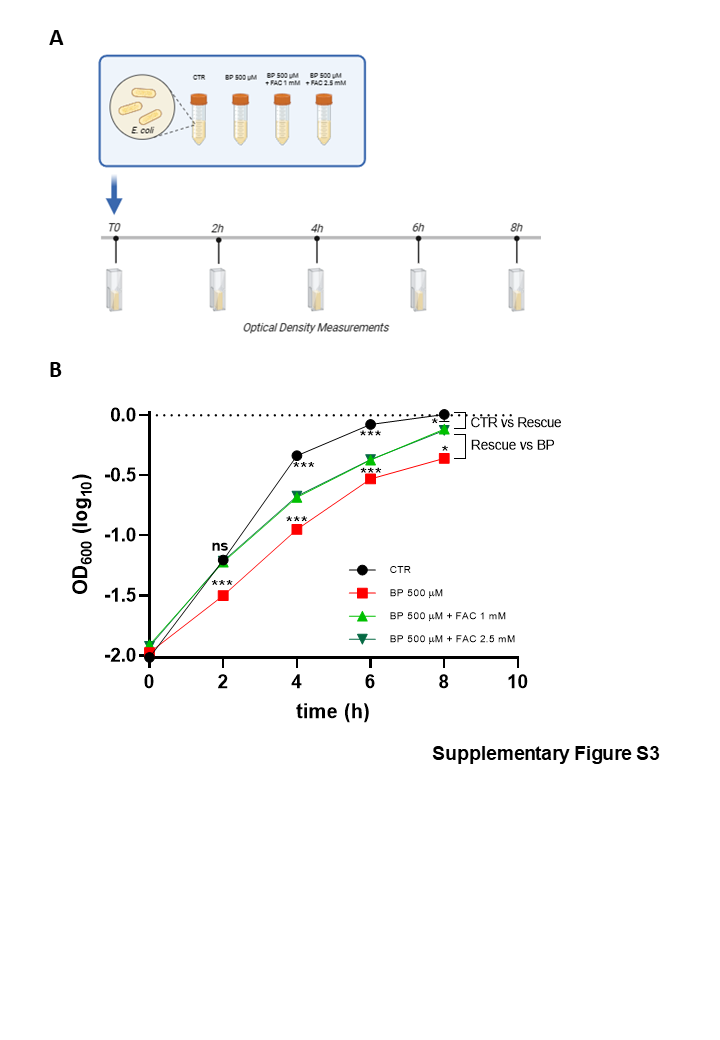


***A.*** Conceptual schematic showing the experimental workflow: bacteria populations were treated with BP and two different concentrations of FAC (1 mM and 2.5 mM) or no treatment (Ctr) for eight hours, taking samples for evaluation each two hours. Optical Density (OD) at 600 nm measurements were evaluated at each timepoint. ***B***. Graph representing dynamics for *E. coli*. Data are indicated using a logarithmic scale (log10) on a line plot depicting the average values between three replicates over time (in hours). Control condition (bacterial growing in normal culture medium) and iron deficiency condition BP 500 µM are compared to FAC 1 mM and FAC 2.5 mM added to iron deficient medium (treated with BP 500 µM). We observed a partial rescue with the addition of FAC to the ID medium. Different conditions are indicated as follows: BP 500 µM colored in red, BP 500 µM + FAC 1 mM colored in light green, BP 500 µM + FAC 2.5 mM in dark green, no treatment (control, CTR) in black. Error bars indicate the standard error of measurement (SEM). Each dot represents the mean value from, at least, three biological replicates. Significance indicated as follows: * p-value < 0.05, ** p-value < 0.005, *** p-value < 0.0005.


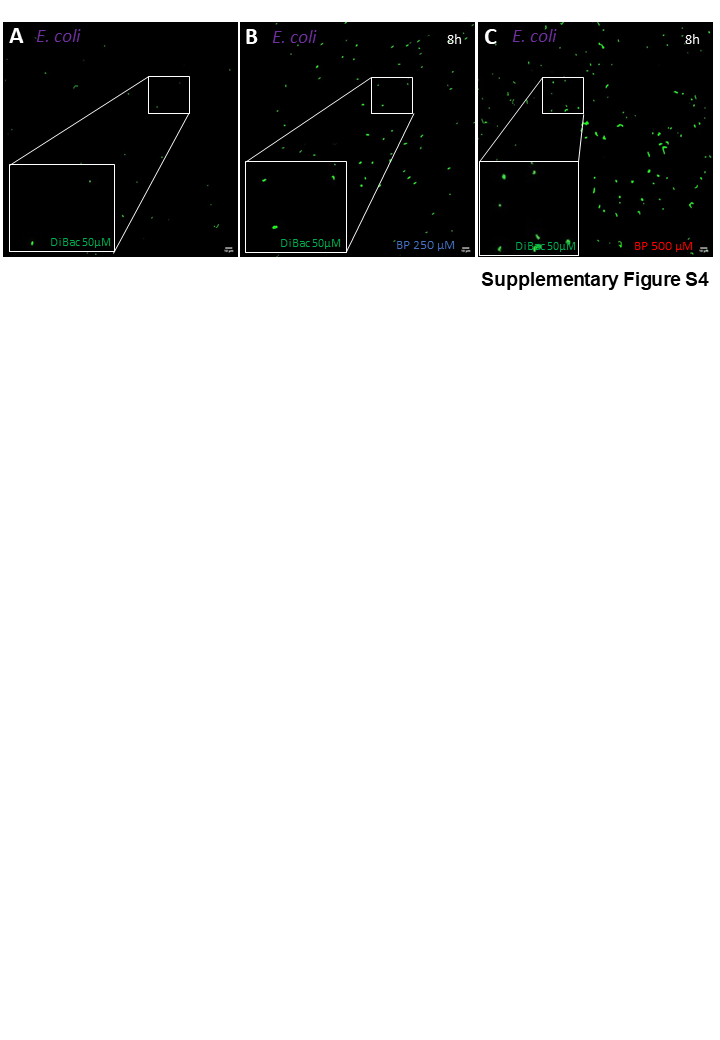


**Supplementary Figure S4**. **Iron deficiency induces changes in *E. coli* V_mem_ potential*.***

***A, B, C.*** Epifluorescence microscope images showing DiBAC-expressing *E. coli* (in green) after 8 hours of BP treatment under a 40X objective for Ctr (top), BP 250 µM (middle) and BP 500 µM (bottom). The images showed a gradual increasing fluorescent intensity with increasing iron deficiency. Scale bar = 10 µm. All pictures have been taken with Leica DMi8 (Leica microsystems; Milano, Italy) inverted microscope and analyzed with ImageJ.

**Supplementary Figure S5**. **Iron deficiency doesn’t induce changes in *L. reuteri* V_mem_ potential*.***


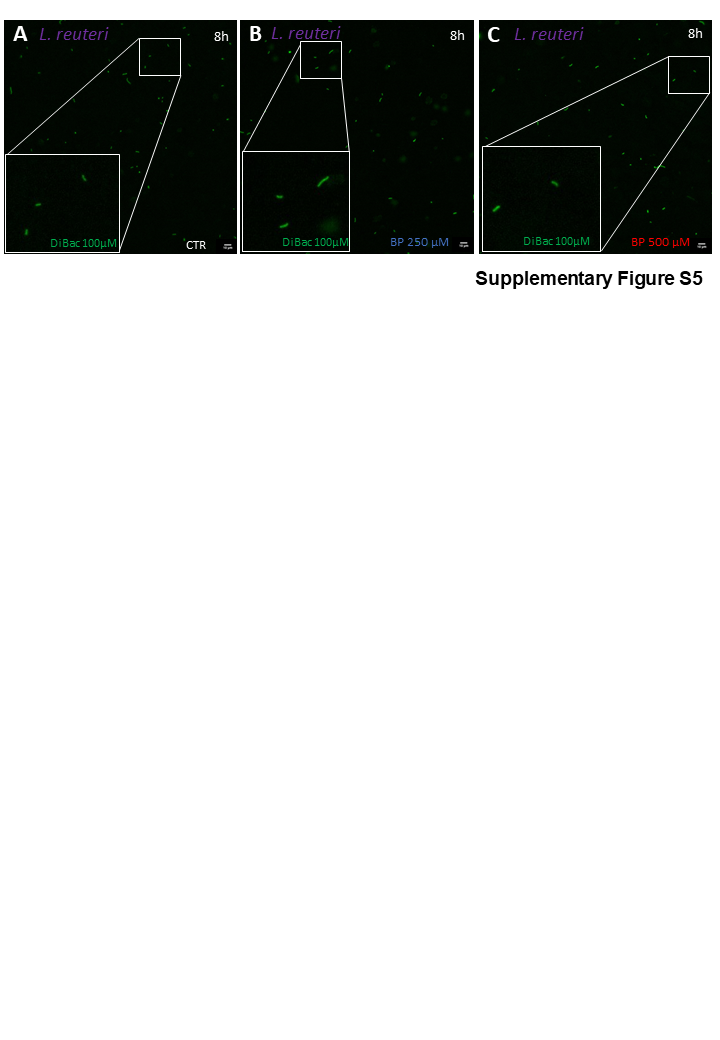


***A, B, C.*** Epifluorescence microscope images showing DiBAC-expressing *L. reuteri* (in green) after 8 hours of BP treatment under a 40X objective for Ctr (top), BP 250 µM (middle) and BP 500 µM (bottom). The images showed no changes in fluorescent intensity with increasing iron deficiency. Scale bar = 10 µm. All pictures have been taken with Leica DMi8 (Leica microsystems; Milano, Italy) inverted microscope and analyzed with ImageJ.

**Supplementary Figure S6**. **Addition of FAC to iron deficiency medium rescues changes in *E. coli* V_mem_ potential*.***


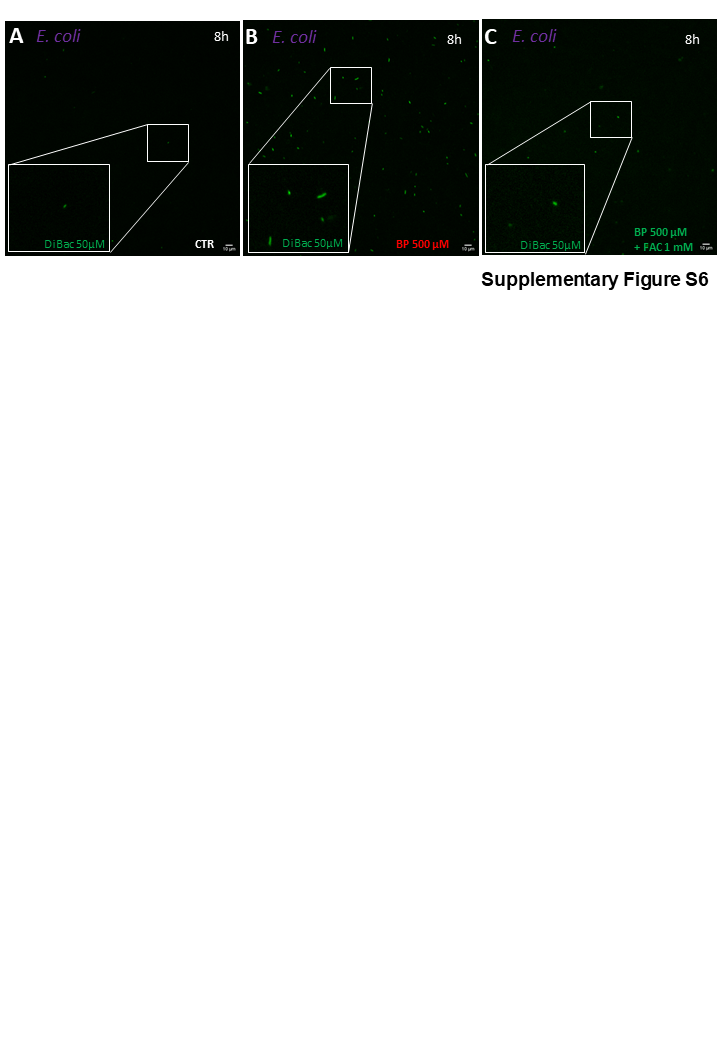


***A, B, C.*** Epifluorescence microscope images showing DiBAC-expressing *E. coli* (in green) after 8 hours of BP treatment under a 40X objective for Ctr (top), BP 500 µM (middle) and BP 500 µM + FAC 1 mM (bottom). The images showed an increasing fluorescent intensity with iron deficiency, then a decrease with iron supplementation to the iron deficient medium. Scale bar = 10 µm. All pictures have been taken with Leica DMi8 (Leica microsystems; Milano, Italy) inverted microscope and analyzed with ImageJ.

**Supplementary Figure S7**. **Fitting of the mathematical model on the experimental data.**
***A, C, E***. Graph depicting how the mathematical model fits our experimental data, plotting population over time. ***B, D, F.*** Logarithmic graph of the fitting of the mathematical model on the experimental data. The blue line indicates the fitting curve for the number of alive bacteria over time while the yellow dots represent the real data obtained from the experiment. The red line indicates the number of dead bacteria over time while the purple dots represent the real data obtained from the experiment. Error bars corresponding to the standard deviation both in normal and logarithmic scale for the abscissas. Cellular populations are taken per mL of medium.


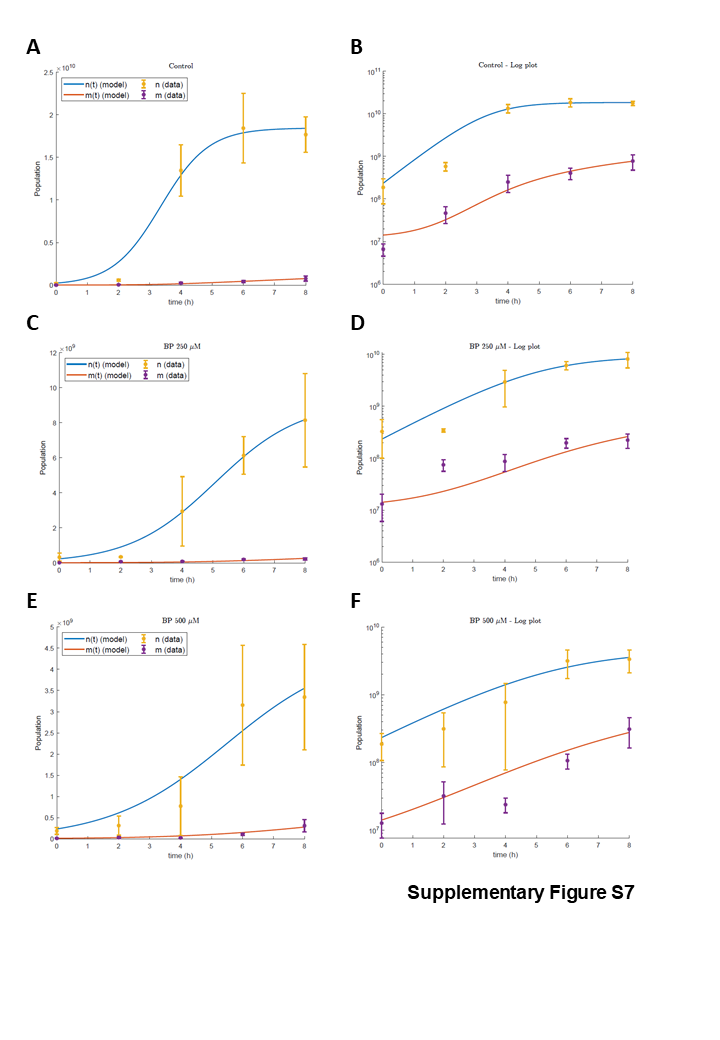


**SUPPLEMENTARY TABLES**

**Supplementary Table S1.** Statistical details for ICP-MS comparisons between experimental groups, for iron, copper (Cu), zinc (Zn) and manganese (Mn) for *E. coli* (left) and *L. reuteri* (right). p values from post-hoc Tukey analysis (after one-way ANOVA p<0.05) are indicated. ns: non-significant differences

|  | ***E. coli*** | | | ***L. reuteri*** | | |
| --- | --- | --- | --- | --- | --- | --- |
| **Time** | **Ctr vs. BP250** | **Ctr vs. BP 500** | **BP 250 vs BP 500** | **Ctr vs. BP250** | **Ctr vs. BP 500** | **BP 250 vs BP 500** |
| Iron | p <0.0001 | p<0.0001 | ns | p=0.0069 | p=0.007 | ns |
| Cu | ns | ns | ns | ns | ns | ns |
| Zn | ns | ns | ns | ns | ns | ns |
| Mn | p =0.009 | p=0.0005 | ns | ns | ns | ns |

**Supplementary Table S2.** Statistical analysis for OD and Cultivability in E. coli among control, BP 250 um and BP 500 uM groups. Statistic F and p values from one-way ANOVA are indicated. ns: non-significant differences

|  | ***E. coli*** | |
| --- | --- | --- |
| **Time** | **OD** | **Cultivability** |
| t = 2 h | F = 10  p =0.012 | ns |
| t = 4 h | F =103.52  p <0.0001 | F =13.81  p <0.01 |
| T=6 h | F =195.23  p < 0.0001 | F =65.01  p < 0.001 |
| t = 8h | F =241.76  p <0.0001 | F =53.99  p <0.0001 |

**Supplementary Table S3.** Statistical details for OD measurements and cultivability comparisons between experimental groups, at each time point for E. coli. Statistic t and p values from post-hoc Tukey analysis (after one-way ANOVA p<0.05) are indicated. ns: non-significant differences

|  | **OD** | | | **Cultivability** | | |
| --- | --- | --- | --- | --- | --- | --- |
| **Time** | **CTR vs. BP250** | **CTR vs. BP 500** | **BP 250 vs BP 500** | **CTR vs. BP250** | **CTR vs. BP 500** | **BP 250 vs BP 500** |
| t = 2 h | t = -3.38  p =0.03 | t = -4.23  p =0.013 | t = -0.85  p =0.688 |  |  |  |
| t = 4 h | t = -6.84  p =0.001 | t = -14.38  p <0.0001 | t = -7.55  p =0.001 | t = -3.05  p =0.03 | t = -5.23  p =0.001 | t = -2.18  p =0.128 |
| T=6 h | t = -7.58  p =0.001 | t = -19.59  p <0.0001 | t = -12.01  p <0.0001 | t = -5.86  p =0.009 | t = -11.39  p =0.001 | t = -6.63  p =0.006 |
| t = 8h | t = -9.67  p <0.0001 | t = -21.94  p <0.0001 | t = -12.26  p <0.0001 | t = -4.30  p =0.007 | t = -10.38  p <0.0001 | t = -5.31  p =0.002 |

| Legend |
| --- |
| P value |
| Coefficient  [CI_95%_ Coefficient] |

**Supplementary Table S4.** Statistical details for Depolarization analysis in *E. coli under different ID conditions*, comparing between experimental groups (after p<0.05 in logistic regresión model)

| **Treatment** | **BP 250** | **BP 500** |
| --- | --- | --- |
| **Control** | <0.0001  1.270  [1.105, 1.435] | <0.0001  2.136  [1.988, 2.283] |
| **BP 250** |  | <0.0001  0.866  [0.740, 0.991] |

**Supplementary Table S5.** Statistical details for Depolarization analysis in *E. coli*, comparing between experimental groups including rescue with FAC (after p<0.05 in logistic regresión model)

| Legend |
| --- |
| P value |
| Coefficient  [CI_95%_ Coefficient] |

| **Treatment** | **BP 500** | **BP 500 + FAC 1mM** |
| --- | --- | --- |
| **Control** | <0.0001  3.702  [3.570, 3.833] | <0.0001  0.9130  [0.726, 1.010] |
| **BP 500** |  | <0.0001  -2.789  [-2.945, -2.632] |

**Supplementary Table S6.** Initial conditions and the parameters used by the mathematical model in *E. coli*. The minimization procedure yields the initial conditions and model parameters detailed in the table below.

| Sample | ***E. coli*** | | |
| --- | --- | --- | --- |
|  | **CTR** | **BP 250 µM** | **BP 500 µM** |
| **τ (mn)** | 31.96 | 59.00 | 80.92 |
| **K** | 1.845e+10 | 9.176e+09 | 4.496e+09 |
| **b** | 1.31 | 0.7271 | 0.5495 |
| **d** | 0.008815 | 0.008823 | 0.02055 |
| **n_0_** | 2.337e+08 | | |
| **‍m_0_** | 1.419e+07 | | |

**Suppl. Table S7. Mathematical model’s fitting with *E. coli* experimental data*.***

The R^2^ values of our fits are the following, in each experimental setting, respectively for live and dead bacteria:

| Sample | *E. coli* | | |
| --- | --- | --- | --- |
| ‍‍ | R^2^ | | |
|  | CTR | BP 250 µM | BP 500 µM |
| ‍live bacteria | 0.9825 | 0.9931 | 0.9078 |
| ‍dead bacteria | 0.9751 | 0.7020 | 0.9167 |
